# Longitudinal study of prevalence and antimicrobial resistance of thermotolerant *Campylobacter* spp. in German organic meat chicken farms

**DOI:** 10.1128/spectrum.01012-26

**Published:** 2026-09-04

**Authors:** Anna Maria Korves-Wilm, Kerstin Stingl, Margret Krieger, Mirjam Grobbel, Bernd-Alois Tenhagen

**Affiliations:** 1 Department Biological Safety, German Federal Institute for Risk Assessment 27652, Berlin, Germany; 2 University of Kassel 9178, Witzenhausen, Germany; Iowa State University of Science and Technology, Ames, Iowa, USA; USDA-ARS Poultry Production and Products Safety Research Unit, Fayetteville, Arkansas, USA; Friedrich-Loeffler-Institut, Federal Research Institute for Animal Health, Thueringen, Germany

**Keywords:** *Campylobacter*, *C. jejuni*, *C. coli*, antimicrobial resistance, organic, broiler

## Abstract

Widespread prevalence of fluoroquinolone-resistant *Campylobacter* spp. in meat chicken is a major concern for public health. We examined the prevalence and antimicrobial resistance of thermotolerant *Campylobacter* spp. in 14 German organic meat chicken farms rearing slow-growing broilers, male layer hybrids, or dual-purpose cockerels in a longitudinal study. Most farms participated with four subsequent flocks. Each flock was sampled three times: approximately 1 month after hatching (before outdoor access), 2 weeks after first outdoor access, and at the end of the fattening period. Strikingly, most flocks were already *Campylobacter*-positive before first outdoor access. At the end of the fattening period, 98% of all flocks were positive, with *C. jejuni* being the predominant species. Antimicrobial resistance of 405 *C. jejuni* and 79 *C. coli* revealed overall high levels of ciprofloxacin and tetracycline resistance but absence of macrolide resistance. Ertapenem resistance was observed in individual farms. Generalized linear mixed models with random factors for farm and flocks within farm were used to assess predictors for the occurrence of ciprofloxacin- and tetracycline-resistant *C. jejuni*. Season, fattening type, and the interaction of sampling time point and fattening type were significant fixed effects. Results for flocks within farms showed clustering effects (ICC_CIP_ = 0.447; ICC_TET_ = 0.412). Whole-genome sequencing of representative isolates confirmed great overall genetic variety, with farm- or fattening type-dependent dissemination of certain multi-locus sequence types. ST21 clonal complex for sequenced *C. jejuni* and ST828 clonal complex for sequenced *C. coli* were most commonly detected.

**IMPORTANCE:**

*Campylobacter* spp. are major foodborne pathogens associated with the consumption and handling of chicken meat. This longitudinal study provides further insight into the prevalence and antimicrobial resistance of thermotolerant *C. jejuni* and *C. coli* in German organic meat chicken farms, a growing sector with strict limitations on antibiotic treatment. We included multiple flocks of farms fattening either slow-growing broilers, male layer hybrids, or dual-purpose cockerels to account for a variety of fattening types. Consequently, we were able to demonstrate significant differences in the occurrence of ciprofloxacin- and tetracycline-resistant *C. jejuni* based on fattening type, sampling time point, and season. Our findings highlight the successful spread and persistence of ciprofloxacin-, tetracycline-, and ertapenem-resistant *C. jejuni* and *C. coli* among all three types of organic meat chicken regardless of the lack of antibiotic treatment.

## INTRODUCTION

Thermotolerant *Campylobacter* spp. (*Campylobacter*) are the leading foodborne pathogens to cause bacterial gastroenteritis in humans, with 168,396 confirmed cases in the European Union in 2024 (1). Symptoms include watery to bloody diarrhea, vomiting, cramps, and fever (2). In rare cases, the infection may lead to autoimmune-mediated neuropathies such as Guillain–Barré syndrome and Miller-Fisher syndrome, or possibly chronic intestinal disease, such as post-infectious inflammatory bowel syndrome (2, 3). The majority of human cases are caused by *C. jejuni* or *C. coli* following the consumption or handling of contaminated chicken meat (1, 4, 5).

Widespread fluoroquinolone resistance among *Campylobacter* is a major concern for public health, as treatment options for severe cases become limited (6). In 2024, resistance of *C. jejuni* and *C. coli* to ciprofloxacin in Germany was high to extremely high in broiler isolates (77.1–90.7 %), following significantly increasing trends since 2014 (7). Similarly, resistance to tetracycline has reached a high to extremely high level (66.7–71.2 %) for *Campylobacter* isolates from German broilers in 2024 (7).

A better understanding of the ecology and transmission of resistant *Campylobacter* within the whole broiler production system is necessary to identify mitigation and control strategies. Both *C. jejuni* and *C. coli* are generally considered commensal bacteria of the chicken intestinal tract, but their role in gut health and chicken welfare is debated (8). *Campylobacter* can usually first be detected in meat chicken flocks 2–3 weeks after hatching (9–11). The exact time of onset in a flock can differ by season, antimicrobial use, husbandry system, and biosecurity measures (10, 12, 13). Free-range and organic husbandry practices are among the risk factors for *Campylobacter* colonization of broiler farms in Germany (14). Studies from Europe and the United States confirm a prevalence of up to 100% in organic flocks compared to conventional flocks (12, 15–19).

Organic meat chicken husbandry in the European Union differs from conventional practices in several aspects that are likely to impact the prevalence and antimicrobial resistance of *Campylobacter*. As per Regulation (EU) No. 2018/848, organic meat chickens must belong to slow-growing breeds or must be slaughtered at a higher age (≥81 days). The allowed maximum stocking density is lower (21 kg/m^2^), flocks are smaller (4,800 birds per group), and free-range farming is mandatory for at least one-third of the chicken’s lifespan, causing increased exposure to wildlife and the surrounding environment. Antimicrobial use and metaphylaxis are not prohibited but limited to one course of treatment in the fattening period (20). For the United States, organic husbandry practices are highly similar in their core principles, but antimicrobial usage is strictly prohibited and leads to the omission of treated animals from the organic label (21). Next to slow-growing broilers, the fattening of alternative chicken types like male layer hybrids and dual-purpose cockerels has become increasingly important with the ban of male chick culling in Germany in 2022 (22, 23).

While an increased prevalence of *Campylobacter* within organic meat chicken flocks has been demonstrated, studies show conflicting results regarding the impact on antimicrobial resistance. In Scandinavian countries with overall lower antimicrobial resistance rates, differences in susceptibility could either not be established (16) or showed higher resistance rates in conventional *Campylobacter* isolates (15). Isolates collected before the ban of fluoroquinolones for poultry production in the United States showed significantly higher overall resistance rates in conventional broiler flocks (17). Following the ban of fluoroquinolones in the United States, a more recent study demonstrated similar susceptibility for all antimicrobials tested but significantly higher tetracycline resistance in fecal isolates from organic broilers at slaughter (24). Better understanding is still needed regarding the characterization of antimicrobial-resistant *Campylobacter* in organic meat chicken farms over the fattening period. Furthermore, the authors are not aware of studies having investigated organically reared dual-purpose cockerels or male layer hybrids, limiting the ability to assess potential influences of chicken genotype.

We therefore designed a longitudinal study to assess the prevalence and antimicrobial resistance of thermotolerant *Campylobacter* in organic meat chicken flocks under natural rearing conditions in Germany. Three different fattening types (slow-growing broilers, male layer hybrids, and dual-purpose cockerels) were compared to account for different chicken genotypes and slaughter ages.

The objectives of our study were to (i) assess the prevalence of thermotolerant *Campylobacter* in organic meat chicken flocks at the end of the indoor period, after frequent outdoor access, and at the age of slaughter, (ii) examine antimicrobial resistance and potential changes in resistance rates depending on sampling time point, fattening type, antimicrobial treatment, and season, and (iii) characterize resistance determinants and perform phylogenetic analysis of representative strains to identify common lineages or clusters.

## MATERIALS AND METHODS

### Study population and sampling time points

As part of the ProBioHuhn project, the farms, providing hatcheries and flock sizes have been described in detail in a previous publication (25). Briefly, 14 small- to medium-sized certified organic meat chicken farms in Germany were acquired for the study. From each farm, either slow-growing broilers (*n* = 5, ISA JA 757 or ISA JA 87 and ICA CY 57), male layer hybrids (*n* = 5, Lohmann Brown-Plus or Dekalb White), or dual-purpose cockerels (*n* = 4, Coffee and Cream) were sampled. Day-old chicks were provided from six different certified organic hatcheries in Germany and the Netherlands. In total, 51 flocks were sampled, with most farms participating with four consecutive flocks. Farm visits were conducted from March 2023 to January 2025, therefore allowing us to account for seasonal variability.

Cloacal swabs (Cary-Blair medium, Süsse Labortechnik GmbH & Co. KG, Gudensberg, Germany) were taken at three different sampling time points (S3–S5) from 10 birds per visit each to assess *Campylobacter* prevalence and antimicrobial resistance at important stages of the fattening period:

1. After approximately 1 month on farm, toward the end of the indoor fattening period (S3).
2. Two weeks after first outdoor access (S4).
3. At the age of slaughter (1–2 weeks before first slaughter or 1–2 weeks after first thinning of the flock) (S5).

Within the ProBioHuhn project, sampling time points S1 and S2 represented meconium samples from day-old chicks upon arrival on farm and boot swabs of the housing compartment after approximately 2 weeks on farm. *Campylobacter* prevalence was not assessed at these time points. Farm 8 specialized in finishing birds originally reared on farms 6 and 9. Consequently, farm 8 was sampled only at S4 and S5, while the source flocks on farms 6 and 9 were sampled at S3, respectively.

All swabs were packaged with a cold pack and sent to the National Reference Laboratory for Antimicrobial Resistance by 24-h express delivery to ensure processing within 72 h of sample collection. A total of 140 pooled swab samples arrived at the laboratory between March 2023 and January 2025. From these samples, five arrived 3–5 days after sampling due to delays in postal service and were therefore omitted from prevalence analysis due to potential false-negative culturing results. Thus, the prevalence of *Campylobacter* for each fattening type was determined from the remaining 135 pooled samples.

### Age ranges of different fattening types per sampling time point

As the growth rate and therefore the duration of the fattening period differed between fattening types, the chickens were of different age ranges at sampling time points S4 and S5 (Table 1).

**TABLE 1:** Mean age and age range in days for each fattening type per sampling time point^*a*^

Fattening type	Mean age at S3 (min.–max.)	Mean age at S4 (min.–max.)	Mean age at S5 (min.–max.)
Slow-growing broilers	31 (25–35)	64 (47–81)	80 (61–100)
Male layer hybrids	32 (27–41)	71 (55–98)	98 (70–119)
Dual-purpose cockerels	31 (26–36)	77 (55–103)	117 (95–138)

^*a*^ S3: end of indoor period, S4: 2 weeks after first outdoor access, and S5: 1–2 weeks before slaughter/after first thinning.

### Additional farm information

Parameters of external and internal biosecurity (other livestock on farm, rodent control, hygiene lock, and cleaning and disinfection measures) were only roughly assessed in our study through questionnaires. They were omitted from statistical analysis due to a lack of sufficient data (incomplete or insufficiently defined answers), but revealed the presence of other livestock and a lack of functional hygiene locks upon entering the barn or mobile house (separate shoes and clothing, facilities for both hand washing and disinfection) in most farms. These data are available in File S1.

### Antimicrobial treatment of flocks

Three flocks received antimicrobial treatment (coccidiostats: toltrazuril, amprolium; antibiotics: tylosin, amoxicillin) during the study to control coccidiosis and necrotizing enteritis (farm 10 flock 2), chronic airway infection with isolation of *Mycoplasma gallisepticum* and *M. synoviae* (farm 10 flock 3), and coccidiosis and *Clostridium* spp. infection (farm 2 flock 4). Time point and doses have been reported previously (25).

### Isolation, identification and storage of isolates

Isolation of *Campylobacter* was performed in accordance with DIN EN ISO 10272-1:2017-09 and the harmonized European Union Reference Laboratory for *Campylobacter* (EURL-*Campylobacter*) protocol for the isolation, identification and storage of *Campylobacter jejuni* and/or *C. coli* for the EU monitoring antimicrobial resistance version 1 (26, 27). All incubation steps were conducted in microaerophilic atmosphere (5% O_2_, 10% CO_2_, and 85% N_2_), which was created by a CO_2_ incubator (Binder GmbH, Tuttlingen, Germany) or in rare exceptions with the use of a GasPak and CO_2_ generator sachet system (Becton Dickinson GmbH, Heidelberg, Germany).

For pool samples, all swabs were resuspended in 50 mL of Bolton bouillon (Bolton bouillon basis and mod. Bolton selective supplement supplemented with 5% laked horse blood, Oxoid, Wesel, Germany) for 15 min at room temperature. Subsequently and before enrichment, 10 µL of the solution was streaked out directly on modified charcoal cefoperazone deoxycholate agar (mCCDA) (CCDA selective supplement, Oxoid and mCCDA basis, Merck KGaA, Darmstadt, Germany) and Butzler agar (Butzler selective supplement and Columbia agar basis, Oxoid; with addition of 5% sheep blood). Both plates were incubated at 41.5 ± 1°C for 44 ± 4 h. In parallel, the bouillon was enriched for 4–6 h at 37°C, followed by 44 ± 4 h at 41.5 ± 1°C. If no *Campylobacter* typical growth was detected on the initial plates of direct streaking, enriched Bolton bouillon was streaked out on mCCDA and Butzler agar, and incubated as described before. If no growth was detected following culturing of the enriched Bolton bouillon, the sample was considered negative for *Campylobacter*.

In case of growth, two presumptive *Campylobacter* colonies per selective medium were isolated on Columbia agar supplemented with 5% sheep blood (Oxoid) and cultured for another 20–24 h at 41.5 ± 1°C. All isolates were assessed under the microscope for typical morphology and vitality (fast movement with intermediate typical tumbling and curved to corkscrew-shaped morphology), positive oxidase activity (Bactident Oxidase, Merck KGaA), positive catalase activity (visible foaming in 3% H_2_O_2_) and no growth on Columbia agar supplemented with 5% sheep blood after 44 ± 4 h at 25°C in aerobic conditions. Isolates were stored in cryobank systems (Mast Diagnostica GmbH, Reinfeld, Germany) at −80°C. For DNA extraction and antimicrobial susceptibility testing, the isolates were re-cultured at 41.5 ± 1°C on Columbia agar supplemented with sheep blood in two steps: first, colony material of one cryo pearl was plated and incubated for 24 h. A subculture was then incubated for another 20 ± 2 h.

DNA used for species identification was extracted from freshly incubated colonies suspended in 400 µL 5% Chelex 100 resin (Bio-Rad Laboratories GmbH, Feldkirchen, Germany) by thermal lysis (95°C, 15 min) and subsequent centrifugation at 14,000 × *g* (4°C, 10 min). The supernatant (2.5 µL) was used in a multiplex real-time PCR to differentiate between *C. jejuni*, *C. coli*, and *C. lari* (28, 29).

### Antimicrobial susceptibility testing

Antimicrobial susceptibility testing was performed through broth microdilution according to the Clinical and Laboratory Standards Institute (CLSI) manuals M45:2015-10 and VET06:2017 on commercial EUCAMP3 plates (Sensititre, Trek Diagnostic Systems Ltd., East Grinstead, United Kingdom) (30, 31). An inoculum of 2–8 × 10^5^ colony-forming units/mL was prepared from freshly grown isolates suspended into cation-adjusted Mueller-Hinton broth (Sensititre, Remel Inc., Lenexa, KS, USA) supplemented with 5% fetal bovine serum (FBS premium, PAN-Biotech GmbH, Aidenbach, Germany). Reference strains of *C. jejuni* (DSM 4688 from the German Collection of Microorganisms and Cell Cultures GmbH) and *C. coli* (2012-70-443-2 from the Technical University of Denmark) were used as quality controls.

Epidemiological cut-off values (ECOFF) according to the European Committee of Antimicrobial Susceptibility Testing (EUCAST) were used as specified in the European Commission Implementing Decision (EU) 2020/1729 and taking into account specifications for AMR reporting from the European Food Safety Authority (EFSA) in case of ertapenem to accurately compare to European national AMR monitoring data (32, 33). The harmonized European testing panel for AMR surveillance according to Commission Implementing Decision (EU) 2020/1729 contained six antibiotics from different antimicrobial classes: chloramphenicol (2–64 mg/L, ECOFF >16 mg/L for *C. jejuni* and *C. coli*), ciprofloxacin (0.12–32 mg/L, ECOFF >0.5 mg/L for *C. jejuni* and *C. coli*), ertapenem (0.12–4 mg/L, ECOFF >0.5 mg/L for *C. jejuni* and *C. coli*), erythromycin (1–512 mg/L, ECOFF >4 for *C. jejuni* and >8 for *C. coli*), gentamicin (0.25–16 mg/L, ECOFF >2 mg/L for *C. jejuni* and *C. coli*), and tetracycline (0.5–64 mg/L, ECOFF >1 mg/L for *C. jejuni* and >2 mg/L for *C. coli*).

Isolates showing a minimum inhibitory concentration (MIC) above ECOFF were defined as resistant to the respective antibiotic. Wild-type isolates not resistant to antibiotics from the EUCAMP3 testing panel were referred to as “phenotypically susceptible” regardless of potential genetic resistance determinants to antimicrobials not phenotypically assessed. Isolates with resistance to substances from three or more antimicrobial classes were considered “multidrug-resistant” (MDR) (34).

### DNA extraction and whole-genome sequencing (WGS)

From every pool sample, one isolate per species and unique resistance profile (including phenotypically susceptible isolates) was chosen for whole-genome sequencing (WGS), resulting in 145 *C. jejuni* and 41 *C. coli* isolates.

Freshly grown isolates were suspended in 1 mL of PBS at OD_600_ of 1.5–2 and centrifuged at 14,000 × *g* at 4°C for 5 min. The supernatant was discarded, and DNA was extracted from the cell pellets using the PureLink Genomic DNA Mini Kit (Invitrogen, Life Technologies Corp., Carlsbad, CA, USA) according to the manufacturer’s protocol. DNA concentration was quantified in a 1x dsDNA BR assay using a Qubit 4 fluorometer (Thermo Fisher Scientific). DNA sequencing libraries were prepared with the Illumina DNA Prep (M) Tagmentation Kit (Illumina Inc., San Diego, CA, USA) for short-read sequencing according to the manufacturer’s instructions, followed by a paired-end sequencing run (2 × 150 bp) on a NextSeq 500 sequencer (Illumina Inc.). Trimming and *de novo* assembly of raw paired-end reads with subsequent quality control was performed through the AQUAMIS pipeline (version 1.4.2) (https://gitlab.com/bfr_bioinformatics/AQUAMIS/) (35). All quality control data for sequenced isolates as well as thresholds are available in Tables S2 and S3.

**TABLE 2:** Summary of *Campylobacter* spp. positive pool samples per sampling time point from slow-growing broiler, male layer hybrids, and dual-purpose cockerels

		No. of *Campylobacter*-positive samples/no. of samples (%)		
Fattening type	Sampling time point^*a*^	Total positive	*C. jejuni^b^*	*C. coli^b^*
Slow-growing broilers	S3	18/20 (90.0)	16/20 (80.0)	4/20 (20.0)
	S4	19/19 (100.0)	19/19 (100.0)	4/19 (21.1)
	S5	15/15 (100.0)	13/15 (86.7)	6/15 (40.0)
Male layer hybrids	S3	8/12 (66.7)	7/12 (58.3)	1/12 (8.3)
	S4	11/13 (84.6)	10/13 (76.9)	3/13 (23.1)
	S5	12/13 (92.3)	12/13 (92.3)	3/13 (23.1)
Dual-purpose cockerels	S3	9/13 (69.2)	9/13 (69.2)	4/13 (30.8)
	S4	14/14 (100.0)	11/14 (78.6)	5/14 (35.7)
	S5	16/16 (100.0)	15/16 (93.8)	4/16 (25.0)

^*a*^ S3: end of indoor period, S4: 2 weeks after first outdoor access, and S5: 1–2 weeks before slaughter/after first thinning.

^*b*^ Four isolates were speciated per sample.

**TABLE 3:** Summary of *C. jejuni* and *C. coli* isolates and resistances per sampling time point from slow-growing broiler, male layer hybrids, and dual-purpose cockerels^*a*^^,*b*^

		No. of resistant *C. jejuni* (%)							No. of resistant *C. coli* (%)						
Fattening type	Sampling time point	Total	CHL	CIP	ERY	ETP	GEN	TET	Total	CHL	CIP	ERY	ETP	GEN	TET
Slow-growing broilers	S3	58	0 (0.0)	29 (50.0)	0 (0.0)	0 (0.0)	0 (0.0)	31 (53.4)	12	0 (0.0)	12 (100.0)	0 (0.0)	7 (58.3)	0 (0.0)	12 (100.0)
	S4	69	0 (0.0)	48 (69.6)	0 (0.0)	5 (7.2)	0 (0.0)	48 (69.6)	7	0 (0.0)	5 (71.4)	0 (0.0)	4 (57.1)	0 (0.0)	6 (85.7)
	S5	46	0 (0.0)	17 (37.0)	0 (0.0)	3 (6.5)	0 (0.0)	19 (41.3)	14	0 (0.0)	9 (64.3)	0 (0.0)	6 (42.9)	0 (0.0)	14 (100.0)
Male layer hybrids	S3	28	0 (0.0)	8 (28.6)	0 (0.0)	8 (28.6)	0 (0.0)	12 (42.9)	3	0 (0.0)	3 (100.0)	0 (0.0)	3 (100.0)	0 (0.0)	0 (0.0)
	S4	37	0 (0.0)	23 (62.2)	0 (0.0)	8 (21.6)	0 (0.0)	16 (43.2)	7	0 (0.0)	5 (71.4)	0 (0.0)	7 (100.0)	0 (0.0)	0 (0.0)
	S5	44	0 (0.0)	26 (59.1)	0 (0.0)	3 (6.8)	0 (0.0)	26 (59.1)	4	0 (0.0)	3 (75.0)	0 (0.0)	3 (75.0)	0 (0.0)	1 (25.0)
Dual-purpose cockerels	S3	31	0 (0.0)	28 (90.3)	0 (0.0)	0 (0.0)	0 (0.0)	28 (90.3)	5	0 (0.0)	5 (100.0)	0 (0.0)	4 (80.0)	0 (0.0)	4 (80.0)
	S4	38	0 (0.0)	27 (71.1)	0 (0.0)	1 (2.6)	0 (0.0)	31 (81.6)	17	0 (0.0)	14 (82.4)	0 (0.0)	11 (64.7)	0 (0.0)	14 (82.4)
	S5	54	0 (0.0)	45 (83.3)	0 (0.0)	0 (0.0)	0 (0.0)	41 (75.9)	10	0 (0.0)	9 (90.0)	0 (0.0)	10 (100.0)	0 (0.0)	10 (100.0)

^*a*^ S3: end of indoor period, S4: 2 weeks after first outdoor access, and S5: 1–2 weeks before slaughter/after first thinning.

^*b*^ CHL, chloramphenicol; CIP, ciprofloxacin; ERY, erythromycin; ETP, ertapenem; GEN, gentamicin; TET, tetracycline.

**TABLE 4:** Pairwise contrasts of fixed effects season, fattening type, sampling time point, and antimicrobial treatment from mixed effects logistic regression^*b*^

		CIP resistance ***C. jejuni***					TET resistance ***C. jejuni***				
		CE	OR	95% CI		*P* ^*a*^	CE	OR	95% CI		*P* ^*a*^
				Lower	Upper				Lower	Upper	
Season	Spring vs Summer	0.3	1.4	0.3	7.2	1.000	−1.2	0.3	0.0	2.0	0.354
	Spring vs Autumn	−2.0	0.1	0.0	1.4	0.119	−1.0	0.4	0.0	3.0	0.583
	Spring vs Winter	0.5	1.6	0.1	17.8	1.000	**2.7**	**15.2**	**1.4**	**164.7**	0.018*
	Summer vs Winter	0.2	1.2	0.2	8.1	1.000	**4.0**	**53.0**	**4.6**	**612.2**	<0.001**
	Autumn vs Summer	**2.3**	**10.3**	**1.8**	**60.3**	0.003*	−0.3	0.7	0.2	2.7	0.654
	Autumn vs Winter	**2.5**	**12.0**	**1.2**	**126.0**	0.032*	**3.7**	**39.6**	**4.0**	**393.7**	<0.001**
Fattening type	Broiler vs Male layer	0.5	1.6	0.1	22.8	0.730	1.4	4.2	0.3	57.8	0.286
	Dual-purpose vs Broiler	2.2	9.2	0.4	193.4	0.206	2.3	9.8	0.5	191.4	0.169
	Dual-purpose vs Male layer	2.7	14.6	0.4	493.6	0.203	**3.7**	**41.0**	**1.3**	**1,262.4**	0.029*
Sampling time point	S3 vs S4	−0.1	0.9	0.2	3.4	1.000	0.6	1.9	0.5	6.4	0.589
	S3 vs S5	0.2	1.2	0.3	4.9	1.000	0.7	2.0	0.6	7.2	0.589
	S4 vs S5	0.3	1.4	0.4	4.6	1.000	0.1	1.1	0.5	2.5	0.867
AB treatment	AB treatment vs No AB	1.1	3.0	0.2	40.3	0.411	0.2	1.2	0.0	38.3	0.904

^*a*^ Sequential Bonferroni adjusted *P*; * significant < 0.05, ** highly significant < 0.001. Significant contrasts are highlighted in bold text.

^*b*^ CE, contrast estimate; CI, confidence interval; CIP, ciprofloxacin; OR, odds ratio; TET, tetracycline. S3: end of indoor period, S4: 2 weeks after first outdoor access, and S5: 1–2 weeks before slaughter/after first thinning.

### Bioinformatic analysis

Multi-locus sequence type (MLST) and antimicrobial resistance genes were analyzed from genome assemblies using the BakCharak pipeline version 3.0.3 (https://gitlab.com/bfr_bioinformatics/bakcharak), which incorporates the mlst module 2.23.0 based on the PubMLST database (last update 27 February 2025) and AMRFinder version 3.12.8 (last update 31 January 2024). All isolates were additionally analyzed for resistance determinants in ResFinder database version 4.4.1 of the Center for Genomic Epidemiology to account for differences between the two databases (36, 37). For both databases, thresholds of 90% identity and 60% coverage were used. Discrepancies were noted in Table S1 , and the closest result based on gene coverage and identity was chosen for analysis. Two new alleles (aspA-782, pgm-1590) and 12 new sequence types (ST14784–ST14788, ST14795–ST14801) were submitted to PubMLST and assigned an official nomenclature (https://pubmlst.org/) (38). Phylogenetic analysis was performed based on core genome (cgMLST) in Ridom SeqSphere+ v10.5.2 (Ridom, Muenster, Germany) with a scheme of 1,343 core loci (39). All assemblies and trimmed reads were uploaded to the GenBank and Sequence Read Archive (SRA) of the National Center for Biotechnology Information (NCBI) under BioProject PRJNA1338716. Additionally, all genome assemblies were submitted to PubMLST.

### Visualization

All graphs were created in GraphPad Prism (version 10.1.2 [324]). Phylogenetic trees were visualized in iTol v7 (40).

### Statistical analyses

All cultivation results for pooled samples and antimicrobial susceptibility of isolates with corresponding metadata were compiled and stored in Microsoft Excel (see Table S1). The data were analyzed using IBM SPSS Statistics 31 version 29 (IBM Corp., Armonk, NY, USA). Generalized linear mixed models (GLMMs) were fitted using a binomial distribution with a logit link to examine the effects of meteorological season (spring: March, April, May; summer: June, July, August; autumn: September, October, November; and winter: December, January, February), sampling time point (combined effect of age and outdoor access as two highly correlated variables), fattening type, the interaction of fattening type and sampling time point, and antimicrobial treatment on the occurrence of ciprofloxacin- or tetracycline-resistant *C. jejuni* isolates.

The antimicrobial resistance of an isolate toward a specific antibiotic was coded as a binary outcome (0 = susceptible, 1 = resistant). As only a few *C. coli* isolates per relevant subgroup were found, antimicrobial resistance in *C. coli* was not statistically analyzed. Similarly, due to a small number of resistant *C. jejuni* isolates, ertapenem resistance was not statistically modeled through mixed-effects logistic regression. All fixed effects were coded categorically. Random intercepts for farm and flock nested within farm were included to account for hierarchical structure (farm/flock/sampling time point) and resulting clustering effects. As flocks for farm 8 were initially reared on either farm 6 or 9, isolates belonging to those flocks were coded to belong to a respective (dummy) flock on their farm of origin at S3. For S4 and S5, those isolates were coded to belong to farm 8. This allowed taking the environment of the birds into account as a random factor for farm at a given time point.

Model fitness was evaluated using corrected Akaike information criterion (AIC), Bayesian information criterion (BIC), and Pseudo-*R*^2^ measures. Additionally, scatter plots of Pearson residuals by predicted probabilities were graphically assessed to ensure that there were no major deviations from the expected distribution. Post hoc pairwise contrasts for main fixed effects and the interaction effect were performed using link function transformation and adjusted for multiple comparisons using sequential Bonferroni. Odds ratios were calculated from pairwise contrast estimates. A *P* value of <0.05 was considered statistically significant. All null models and final models are available in File S2.

## RESULTS

### Prevalence and isolation of thermotolerant *Campylobacter*

A total of 122/135 pooled samples (90.4%) were positive for *Campylobacter* (Table 2). For 84.4% (114/135) of samples, *Campylobacter* could already be cultivated on either mCCDA or Butzler medium by direct plating. Parallel enrichment in Bolton broth did slightly increase detection rates (5.9%, 8/135) and led to easier subcultivation of *Campylobacter* colonies within background microbiota in some cases.

Overall, *Campylobacter* could be detected on all fourteen farms, with 98% (49/50) of flocks being positive at the end of fattening (S5). One flock was excluded from the calculation of the overall flock prevalence, as this flock was only sampled once at S3 and might have shown positive results later in the fattening period.

Only one flock of male layer hybrids from farm 12 remained negative for all three sampling time points. The majority of flocks (66.7–90.0 %) were already positive for *Campylobacte*r at the first sampling time point (S3). In all three fattening types and at all sampling time points, the proportion of samples positive for *C. jejuni* (58.3–100%) was higher than for *C. coli* (8.3–40.0%). From 17.8% (24/135) of samples, both *C. jejuni* and *C. coli* were isolated.

In total, 484 *Campylobacter* were isolated (Table 3). Of these, 83.7% (405/484) were *C. jejuni* and 16.3% (79/484) *C. coli*.

### Antimicrobial resistance of *C. jejuni* and *C. coli*

All 405 *C. jejuni* isolates and all 79 *C. coli* isolates were submitted to antimicrobial susceptibility testing.

**Fig 1:**
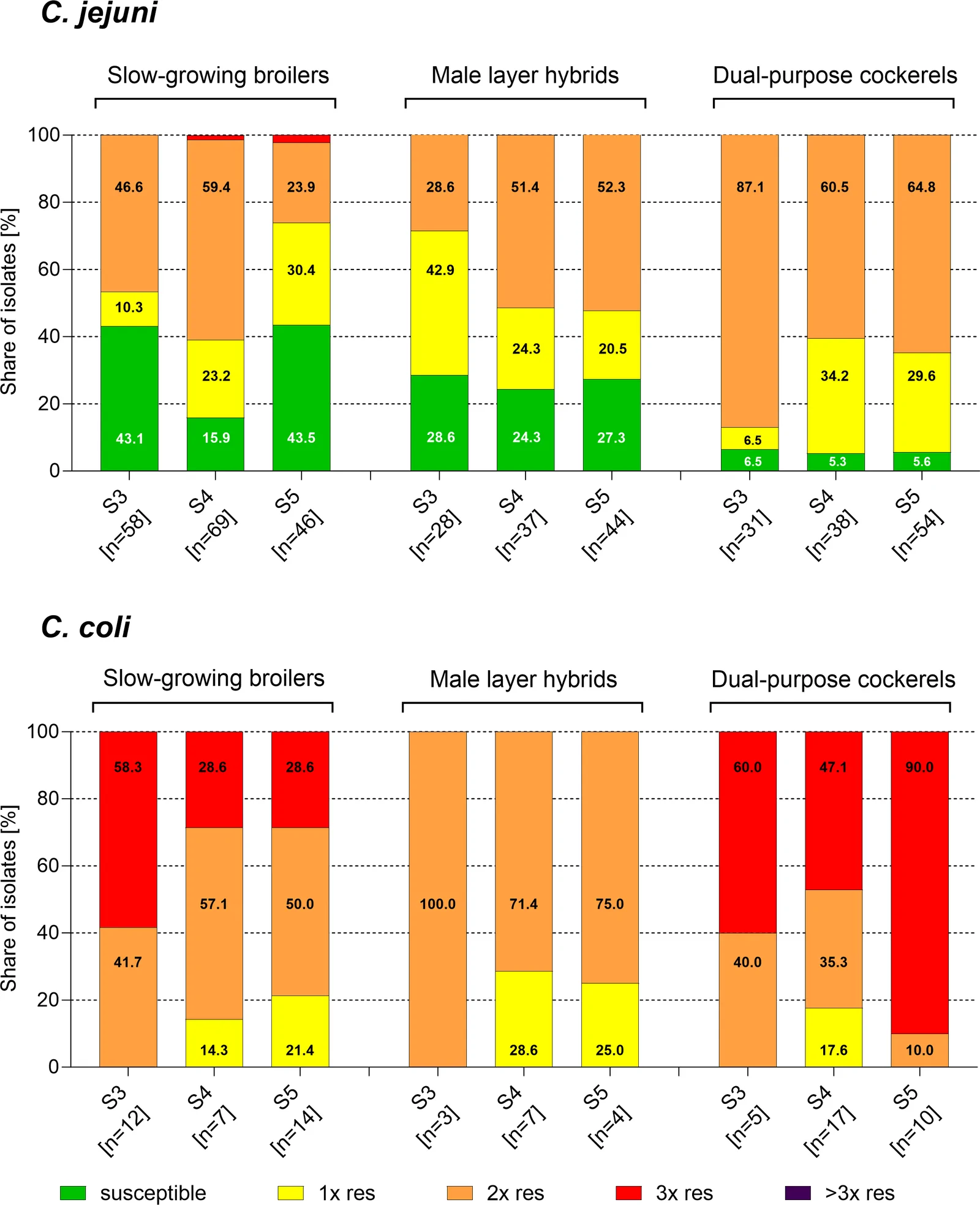
Share (%) of resistant *C. jejuni* and *C. coli* isolates per fattening type and sampling time points S3–S5. Resistance of four *Campylobacter* spp. isolates per pooled sample (total of 405 *C. jejuni* and 79 *C. coli*) was analyzed through broth microdilution on a harmonized European testing panel (EUCAMP3) containing six antimicrobial classes. The share of isolates resistant to 1 (yellow) to >3 (dark violet) different antimicrobial classes is represented as a stacked bar chart. Isolates not phenotypically resistant according to the testing panel are considered phenotypically susceptible (green).

Figure 1 depicts the shares of susceptible and multidrug-resistant isolates. Overall, the percentage of susceptible *C. jejuni* isolates was highest in isolates from slow-growing broilers at S3 and S5, but showed a noticeable decrease to 15.9% at S4 after animals were initially allowed outdoor access. In comparison, the lower share of susceptible isolates in both male layer hybrids and dual-purpose cockerels remained stable over the course of fattening. The majority of resistant *C. jejuni* isolates was resistant toward one or two antimicrobial classes in all three chicken types. MDR *C. jejuni* isolates were only detected in slow-growing broilers at S4 and S5 with 1.5% and 2.2%.

No susceptible isolates and higher shares of MDR isolates could be detected in *C. coli*. Depending on sampling time point, 28.6–58.3 % of isolates from slow-growing broilers and 47.1–90.0 % of isolates from dual-purpose cockerels were resistant to three antimicrobial classes.

None of the *C. jejuni* or *C. coli* isolates were resistant to chloramphenicol, erythromycin, or gentamicin (Table 3). Isolates of *C. jejuni* were primarily resistant to ciprofloxacin (CIP) (9.1%, 37/405), tetracycline (TET) (11.9%, 48/405), or in most cases a combination of both (49.4%, 200/405). Ertapenem (ETP) resistance was overall less prevalent in *C. jejuni* with a total of 28/405 (6.9 %) isolates. Most ETP-resistant isolates were retrieved from male layer hybrids (67.9%, 19/28 ETP-R *C. jejuni*), with the majority originating from farm 13 (53.6 %; 15/28 ETP-R *C. jejuni*).

The majority of *C. coli* were multidrug-resistant with a CIP-TET-ETP profile (41.8%, 33/79). Otherwise, combined resistance profiles of CIP-TET (20.3%, 16/79), CIP-ETP (16.5%, 13/79), TET-ETP (8.9%, 7/79) were more common than resistance to a single antibiotic. Compared to *C. jejuni*, *C. coli* isolates showed higher rates of resistance to ETP with a total of 55/79 (69.6 %) isolates. ETP-resistant *C. coli* were mostly isolated from dual-purpose cockerels (45.5%, 25/55 ETP-R *C. coli*) and slow-growing broilers (30.9%, 17/55 ETP-R *C. coli*). Most of the ETP-R *C. coli* from dual-purpose cockerels (84.0%, 21/25) originated from farm 4 (80.0 %, 12/15 total *C. coli*) and farm 6 (90.0 %, 9/10 total *C. coli*). The majority of all *C. coli* isolated from male layer hybrids were resistant to ETP (92.9%, 13/14 total *C. coli*).

### Mixed-effects logistic regression of CIP and TET resistance in *C. jejuni*

The full generalized linear mixed models with random and fixed effects explained 73.3% and 75.3% of variance for CIP and TET resistance, based on conditional Pseudo-*R*^2^ measures. In both models, flocks within a farm (conditional ICC_CIP_ = 0.447; conditional ICC_TET_ = 0.412) showed higher clustering effects than on the farm level (conditional ICC_CIP_ = 0.098; conditional ICC_TET_ = 0.087). Overall percentage of accuracy in isolate classification was 92.6% (CIP) and 90.9% (TET) with a sensitivity of 96.4% (CIP, TET) and a specificity of 86.4% (CIP) and 81.7% (TET), respectively.

The final corrected models for fixed effects were significant (*F*_CIP_ (12, 392) = 3.308, *P* < 0.001; *F*_TET_ (12, 392) = 3.960, *P* < 0.001). For the occurrence of CIP-R *C. jejuni* isolates, season (*F* (3, 392) = 5.170, *P* = 0.002) and the interaction between fattening type and sampling time point (*F* (4, 392) = 4.619, *P* = 0.001) were significant effects. For the occurrence of TET-R isolates, season (*F* (3, 392) = 6.828, *P* < 0.001), fattening type (*F* (2, 392) = 3.477, *P* = 0.032) and the interaction between fattening type and sampling time point (*F* (4, 392) = 8.898, *P* < 0.001) were significant effects. Detailed fixed effects and fixed coefficients tables are provided in File S2.

Post hoc pairwise contrasts of seasons show that the odds of CIP resistance were higher in autumn compared to summer (*P* = 0.003) and winter (*P* = 0.032) (Table 4). TET-resistant *C. jejuni* were more likely to occur in spring (*P* = 0.018), summer (*P* < 0.001) or autumn (*P* < 0.001) compared to winter. While there was no significant effect of fattening type for CIP resistance, it was almost 41 times more likely to isolate TET-resistant *C. jejuni* from dual-purpose cockerels than from male layer hybrids. Neither sampling time point nor antimicrobial treatment had significant global associations with CIP and TET resistance.

**Fig 2:**
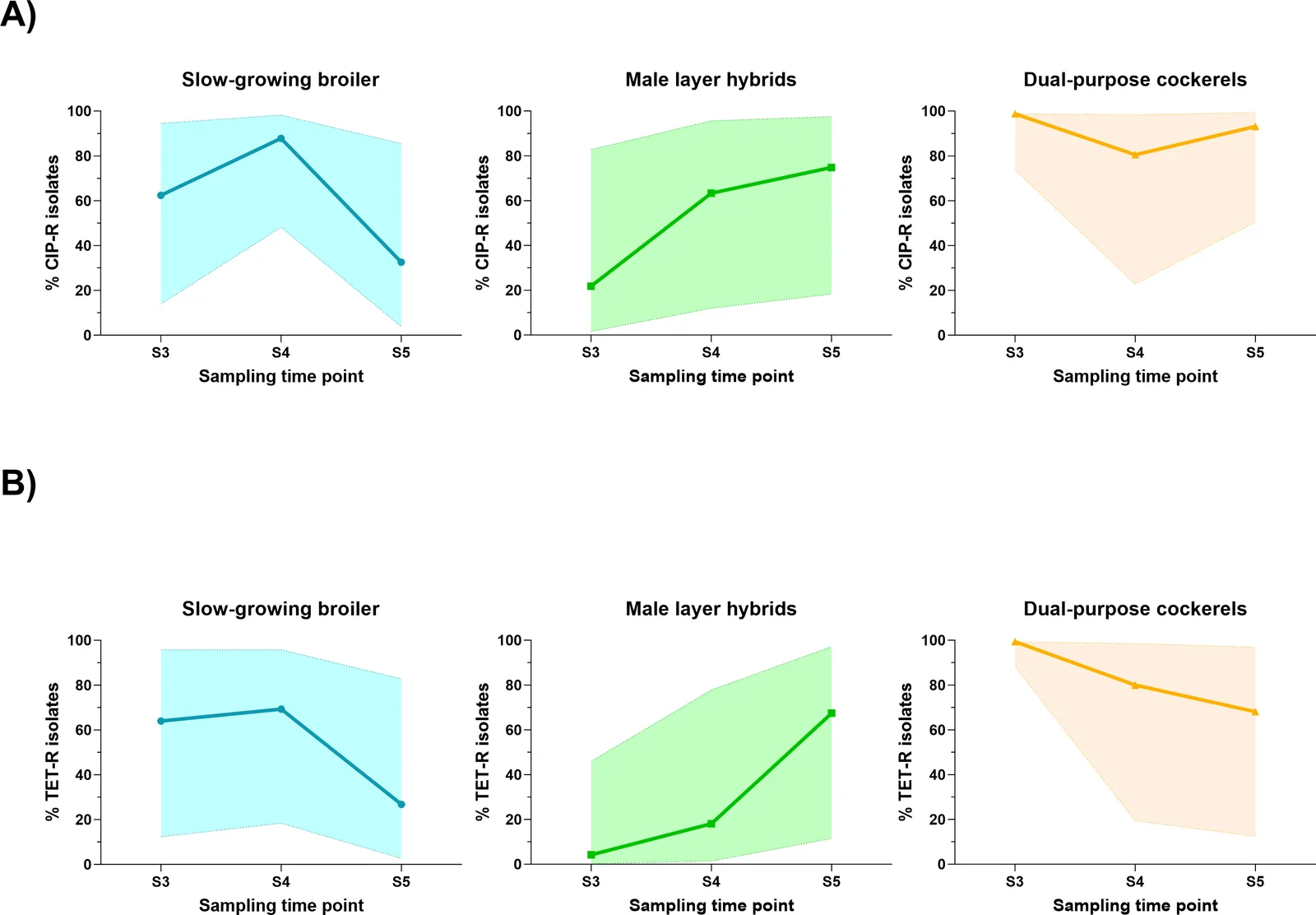
Estimated marginal means and 95% confidence intervals of ciprofloxacin (**A**) and tetracycline (**B**) resistance determined through mixed-effects logistic regression models for *C. jejuni*. Estimated marginal means (EMMs) for CIP- and TET-resistant *C. jejuni* isolates were calculated for the final generalized linear mixed models in SPSS v.31 to illustrate the expected values with 95% confidence intervals for each sampling time point within different fattening types as other fixed effects are held constant.

Line graphs of estimated marginal means and 95% intervals for CIP and TET resistance over the fattening period in different fattening types are depicted in Fig. 2. Between fattening types, significant differences were only observed at S3, where dual-purpose cockerels had significantly higher odds for CIP resistance (*P* = 0.019) than male layer hybrids (Table 5). This effect was even more pronounced for TET resistance at S3, where dual-purpose cockerels had higher odds for TET-resistant isolates than male layer hybrids (*P* < 0.001) and slow-growing broilers (*P* = 0.012). Additionally, TET-resistance was more likely to occur in slow-growing broilers than in male layer hybrids (*P* = 0.016). Within fattening types, significant differences for the occurrence of resistant *C. jejuni* isolates were observed between sampling time points (Table 6). In slow-growing broilers, TET and CIP resistance was around 6 (*P* = 0.032) to 15 times (*P* = 0.006) more likely at S4, 2 weeks after first outdoor access, compared to the end of the fattening period at S5. In male layer hybrids, the odds of retrieving TET-resistant isolates increased over the fattening periods. While the OR between S3 and S4 was not significant (*P* = 0.051), TET-resistant isolates were significantly more likely to occur at the end of the fattening period (S5) compared to S3 (*P* < 0.001) or S4 (*P* = 0.006). In contrast, this effect was reversed in dual-purpose cockerels, where the odds of retrieving TET-resistant isolates were higher at S3 compared to S4 (*P* = 0.005) and S5 (*P* = 0.001).

**TABLE 5:** Pairwise contrasts of fattening types at sampling time points S3-S5 from mixed effects logistic regression^*b*^

		CIP resistance *C. jejuni*					TET resistance *C. jejuni*				
Sampling time point	Fattening types	CE	OR	95% CI		*P* ^*a*^	CE	OR	95% CI		*P* ^*a*^
				Lower	Upper				Lower	Upper	
S3	Broiler vs Male layer	1.8	6.0	0.3	134.1	0.261	**3.7**	**40.3**	**2.0**	**820.8**	0.016*
	Dual-purpose vs Broiler	3.9	48.4	0.8	3,087.2	0.073	**4.5**	**91.3**	**2.3**	**3,617.0**	0.012*
	Dual-purpose vs Male layer	**5.7**	**288.4**	**2.0**	**41,719.5**	0.019*	**8.2**	**3,678.6**	**43.3**	**312,582.6**	<0.001**
S4	Broiler vs Male layer	1.4	4.2	0.1	151.6	1.000	2.3	10.2	0.4	263.8	0.218
	Dual-purpose vs Broiler	−0.6	0.6	0.0	14.2	1.000	0.6	1.8	0.1	32.3	0.697
	Dual-purpose vs Male layer	0.9	2.4	0.1	79.1	1.000	2.9	18.1	0.4	782.3	0.195
S5	Broiler vs Male layer	−1.8	0.2	0.0	4.7	0.449	−1.7	0.2	0.0	5.6	0.645
	Dual-purpose vs Broiler	3.3	27.8	0.7	1,049.4	0.085	1.8	5.8	0.2	176.9	0.645
	Dual-purpose vs Male layer	1.5	4.5	0.2	119.7	0.449	0.0	1.0	0.1	19.9	0.984

^*a*^ Sequential Bonferroni adjusted *P*; * significant < 0.05, ** highly significant < 0.001. Significant contrasts are highlighted in bold text.

^*b*^ CE, contrast estimate; CI, confidence interval; CIP, ciprofloxacin; OR, odds ratio; TET, tetracycline. S3: end of indoor period, S4: 2 weeks after first outdoor access, and S5: 1–2 weeks before slaughter/after first thinning.

**TABLE 6:** Pairwise contrasts of sampling time points within fattening types from mixed effects logistic regression^*b*^

		CIP resistance *C. jejuni*					TET resistance *C. jejuni*				
Fattening types	Sampling time point	CE	OR	95% CI		*P* ^*a*^	CE	OR	95% CI		*P* ^*a*^
				Lower	Upper				Lower	Upper	
Broiler	S3 vs S4	−1.5	0.2	0.0	1.2	0.095	−0.2	0.8	0.2	3.0	0.730
	S3 vs S5	1.2	3.4	0.8	15.3	0.105	1.6	4.9	1.0	24.9	0.059
	S4 vs S5	**2.7**	**14.9**	**1.8**	**121.6**	0.006*	**1.8**	**6.2**	**1.1**	**33.9**	0.032*
Male layer	S3 vs S4	−1.8	0.2	0.0	1.6	0.144	−1.6	0.2	0.0	1.0	0.051
	S5 vs S3	2.4	10.6	0.9	119.9	0.059	**3.8**	**46.8**	**4.8**	**455.4**	<0.001**
	S5 vs S4	0.5	1.7	0.4	7.3	0.461	**2.2**	**9.4**	**1.8**	**49.7**	0.006*
Dual-purpose	S3 vs S4	3.0	19.5	0.4	909.3	0.191	**3.7**	**40.6**	**2.6**	**644.1**	0.005*
	S3 vs S5	1.8	6.0	0.2	169.4	0.428	**4.3**	**76.3**	**4.7**	**1,241.9**	0.001*
	S4 vs S5	−1.2	0.3	0.0	2.6	0.428	0.6	1.9	0.4	9.1	0.432

^*a*^ Sequential Bonferroni adjusted *P*; * significant < 0.05, ** highly significant < 0.001. Significant contrasts are highlighted in bold text.

^*b*^ CE, contrast estimate; CI, confidence interval; CIP, ciprofloxacin; OR, odds ratio; TET, tetracycline. S3: end of indoor period, S4: 2 weeks after first outdoor access, and S5: 1–2 weeks before slaughter/after first thinning.

### Genetic diversity of *Campylobacter*

Based on phenotypical antimicrobial susceptibility, one isolate per species and unique resistance profile (including phenotypically susceptible isolates) was chosen for whole-genome sequencing from each pool sample, resulting in 145 *C. jejuni* and *41 C. coli* isolates.

**Fig 3:**
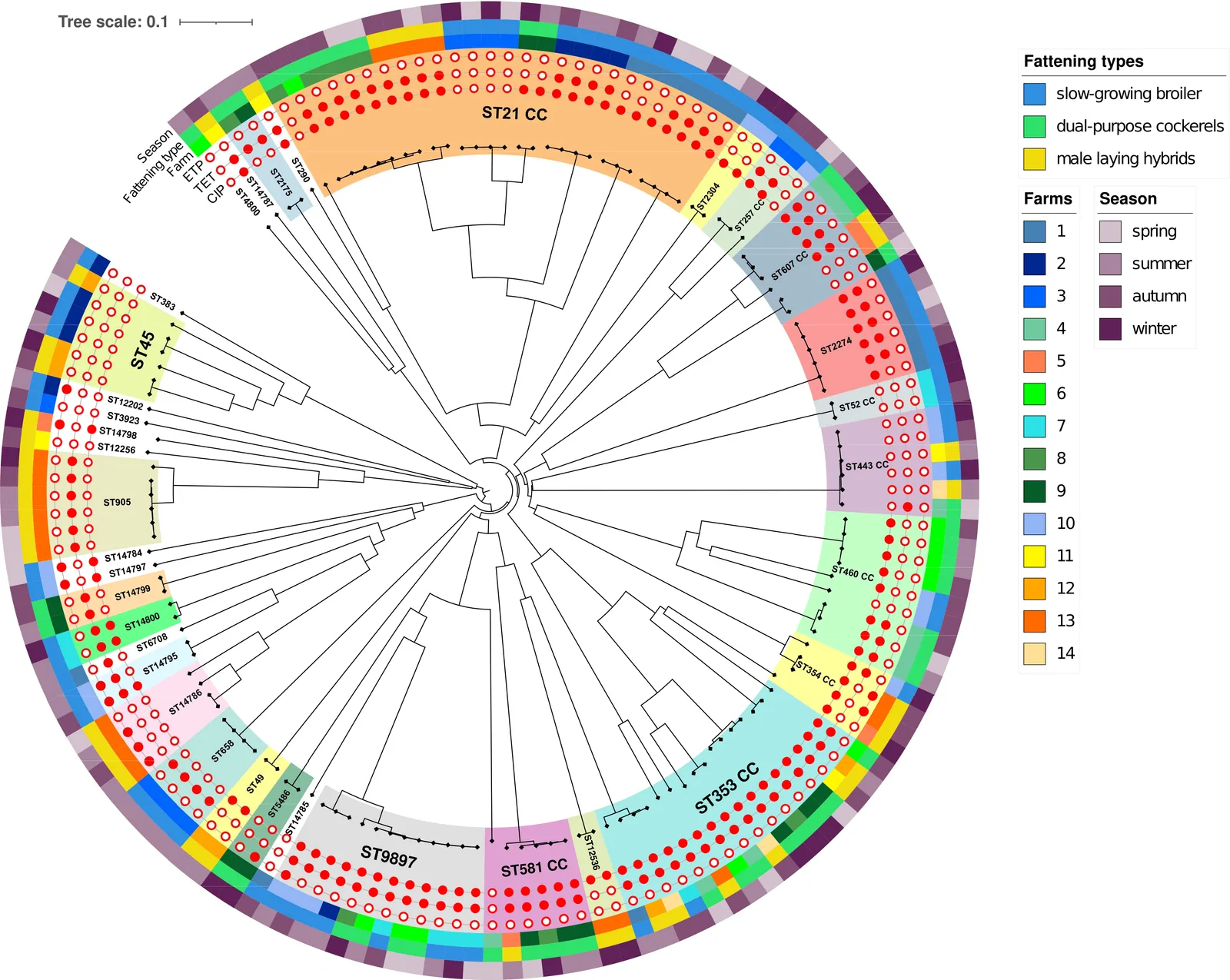
Phylogenetic tree of *C. jejuni* (*n* = 145). A neighborhood joining tree was calculated based on core genome multi-locus sequence type (cgMLST) with Ridom SeqSphere+  v10.5.2 and constructed and annotated in iTol v7 as a midpoint-rooted circular tree. Isolates of different multi-locus sequence types (MLSTs) belonging to the same clonal complex are labeled with the respective clonal complex and highlighted in a colored range. Otherwise, isolates are labeled and highlighted according to their shared MLST. Fattening types, farms, and sampling season are represented as colored strips. Phenotypical resistance of isolates to ertapenem (ETP), tetracycline (TET), and ciprofloxacin (CIP) based on broth microdilution is indicated through filled red circles.

The *C. jejuni* population exhibited great genetic variety with 50 different MLST. Most isolates (64.8%, 94/145) could be assigned to 1 of the 15 different common clonal complexes (CCs) (Fig. 3). The largest number of isolates was attributed to ST21-CC (18.6%, 27/145). Within this complex, ST50 constituted the most commonly identified MLST overall with 13.1% (19/145). ST21-CC was identified among isolates from all three fattening types. Interestingly, the second largest group of isolates belonged to ST353-CC (11.0%, 16/145), which could instead mostly be isolated from dual-purpose cockerels and male layer hybrids. The third and fourth largest groups belonged to ST9897 (8.3%, 12/145) and ST460-CC (6.2%, 9/145), both mostly isolated from slow-growing broilers and dual-purpose cockerels. In these four most common MLST and clonal complexes, isolates predominantly exhibited a CIP-TET resistance profile (87.5%, 49/56).

Following the detection of clonal complexes across the fattening types, the most common MLST within each fattening type differed. ST50 was most prevalent in slow-growing broilers (12.9%, 8/62 isolates) and dual-purpose cockerels (15.9%, 7/44 isolates), while ST905 was most prevalent in male layer hybrids (15.4%, 6/39), but could only be isolated from farm 13. Several other MLSTs were distinct to certain farms, and closely related isolates were repeatedly isolated at several sampling time points and across subsequent flocks, such as ST2274 from slow-growing broiler farm 1 or ST14786 from male layer hybrid farm 13.

Ten clusters of isolates with a cgMLST allele difference ≤10 spanning different farms were detected and examined for putative epidemiological links (File S3). Three of these clusters showed clonal ST2175, ST2153, and ST10025 isolates detected in different flocks and sampling time points on farms 8 and 9. Those farms are epidemiologically linked, as farm 8 finishes chickens reared on farm 9. Similarly, farms 6 and 8 are connected by the same means, explaining the circulation of clonal ST50 isolates on both farms. Closely related isolates were also detected on farm 13 without known connections to farms 6, 8, and 9. Another four clusters spanned multiple farms without any established connections and throughout different seasons. Two clusters of ST607 and ST7355 isolates could be traced back to the same hatchery providing chicks for the respective flocks but were isolated on different farms only after first outdoor access had been granted.

**Fig 4:**
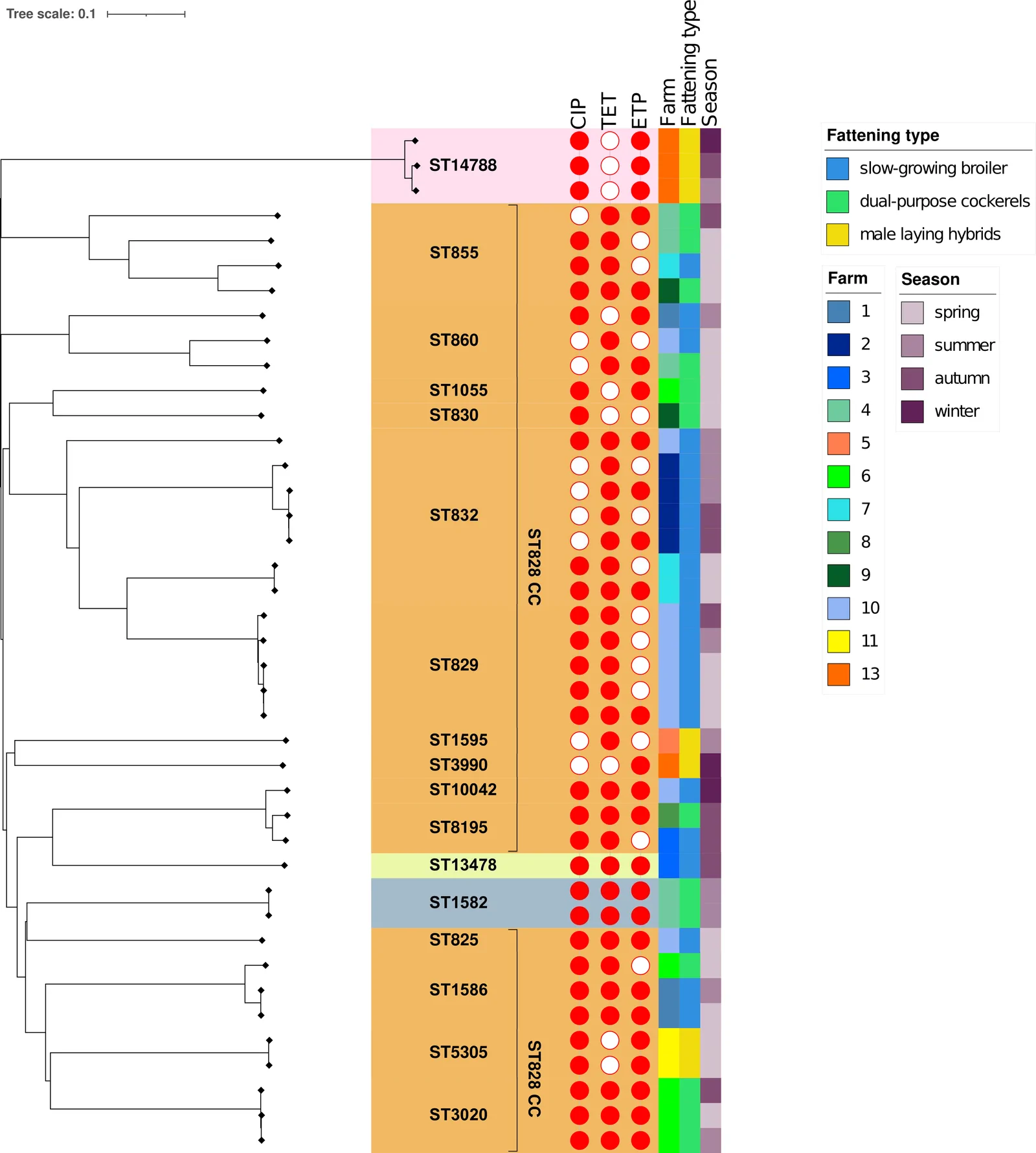
Phylogenetic tree of *C. coli* (*n* = 41). A neighborhood joining tree was calculated based on core genome multi-locus sequence type (cgMLST) with Ridom SeqSphere+  v10.5.2 and constructed and annotated in iTol v7. Isolates of different multi-locus sequence types (MLSTs) belonging to the same clonal complex are labeled with the respective clonal complex and highlighted in a colored range. Otherwise, isolates are labeled and highlighted according to their shared MLST. Fattening types, farms, and sampling season are represented as colored strips. Phenotypical resistance of isolates to ertapenem (ETP), tetracycline (TET), and ciprofloxacin (CIP) based on broth microdilution is indicated through filled red circles.

The majority of *C. coli* isolates were assigned to ST828-CC (85.4%, 35/41) (Fig. 4). Isolates not assigned to ST828-CC belonged to novel ST14788 (7.3%, 3/41), ST1582 (4.9%, 2/41), or ST13478 (2.4%, 1/41). Among isolates assigned to ST828-CC, ST832 (20.0%, 7/35) and ST829 (14.3%, 5/35, all farm 10) were the most prevalent and isolated exclusively from farms fattening slow-growing broilers. In contrast, the most prevalent MLST in dual-purpose cockerels were ST3020 and ST855 (both 7.3%, 3/41), with ST3020 being isolated exclusively from farm 6. In male layer hybrids, ST14788 (7.3%, 3/41, all farm 13) and ST5305 (4.9%, 2/41, all farm 11) were the most prevalent MLST.

### Resistance determinants and phenotypical resistance in *Campylobacter*

**Fig 5:**
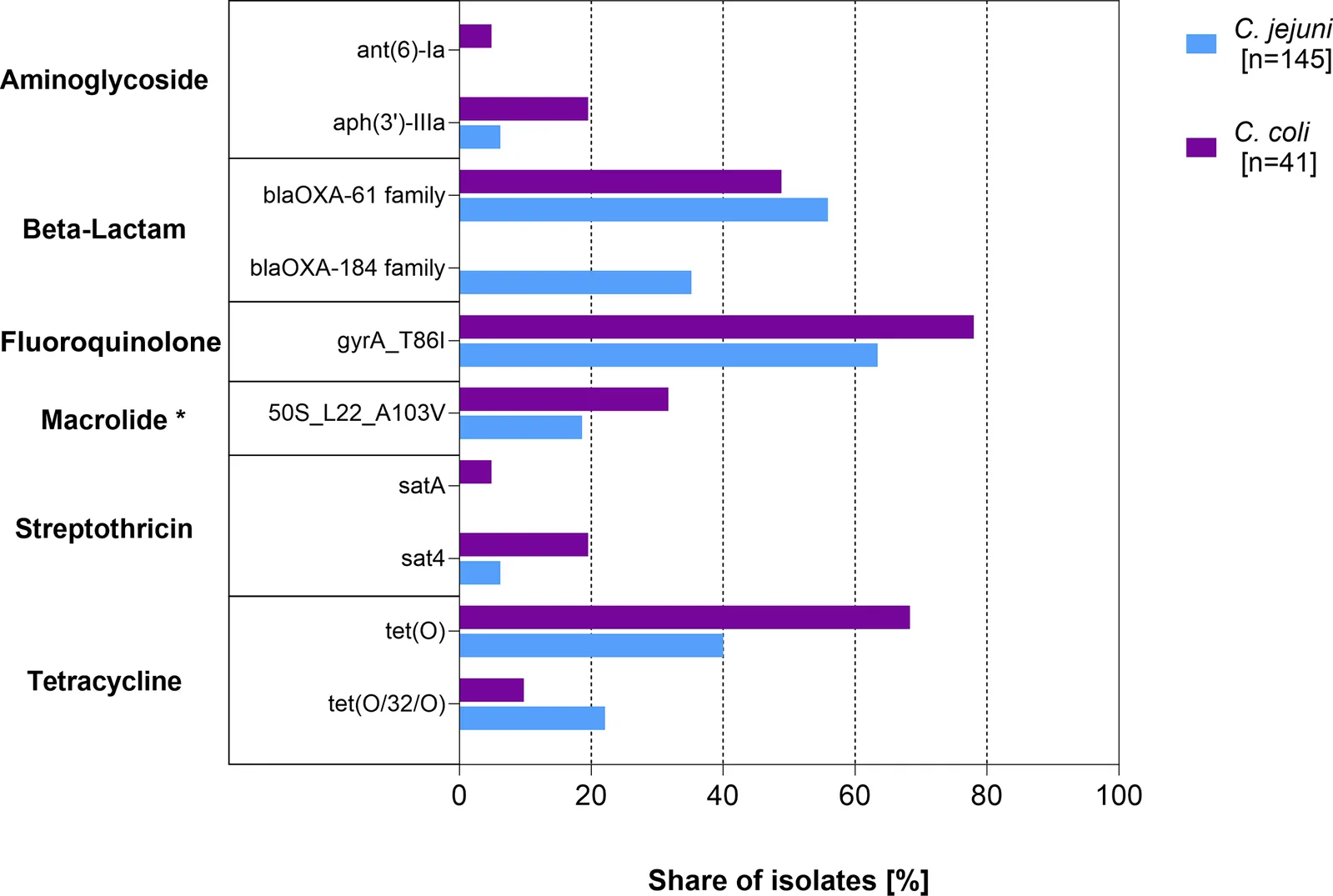
Share (%) of genetic resistance determinants from sequenced *C. jejuni* and *C. coli* isolates. Resistance genes and chromosomal point mutations of short-read sequenced *C. jejuni* (*n* = 145) and *C. coli* (*n* = 41) were determined through AMRFinder and ResFinder and represented as a vertical bar chart. Genetic resistance determinants were sorted according to the antibiotic class to which resistance is conferred according to database annotations. * Macrolide: Isolates with 50S_L22_A103V point mutation did not show phenotypical resistance to macrolides.

Resistance genes and chromosomal point mutations associated with antimicrobial resistance were identified through the AMRFinder and ResFinder databases for sequenced isolates (Fig. 5). Phenotype and resistance determinants for each isolate are available through Table S1.

WGS included 30 phenotypically susceptible *C. jejuni* isolates. In two of them, no resistance determinants were found. Otherwise, beta-lactamase genes of the *bla*_OXA-61_ family were prevalent among isolates, with 48.8% *C. coli* (20/41) and 55.9% *C. jejuni* (81/145) carrying at least one gene belonging to this family. Another 35.2% of *C. jejuni* isolates (51/145) carried genes belonging to the *bla*_OXA-184_ family. As resistance to beta-lactam antibiotics was not tested within the EUCAMP3 panel, these genes were also identified in phenotypically susceptible isolates.

None of the isolates showed phenotypical resistance to erythromycin, yet the A103V point mutation of the 50S ribosomal protein L22 was identified in 18.6% (27/145) of *C. jejuni* and 31.7% (13/41) of *C. coli* isolates. None of the isolates showed phenotypical resistance to gentamicin, but genes conferring resistance to other aminoglycosides could be identified in 24.4% (10/41) of *C. coli* and 6.2% (9/145) of *C. jejuni*. All but one phenotypically CIP-resistant isolates were associated with chromosomal point mutation *gyrA* p. T86I and showed MICs ≥ 8 mg/L. In one CIP-resistant isolate, MIC was slightly above ECOFF (1 mg/L) without detection of *gyrA* p. T86I or other point mutations and, therefore, might be caused by increased efflux pump activity. All phenotypically TET-resistant isolates carried either the *tet*(O) gene or the mosaic variant *tet*(O/32/O). The latter could only be identified in ResFinder, whereas AMRFinder identified them as *tet*(O) with 93.43–93.58% identity. Three isolates phenotypically susceptible to TET still carried the *tet*(O) gene. In these isolates, the *tet*(O) gene was not functional due to C1576T (*n* = 2) or C1420T point (*n* = 1) mutations leading to insertion of a premature stop codon. ETP resistance in *Campylobacter* isolates could not be associated with any distinct resistance determinants defined in the AMRFinder or ResFinder database. No clear association with the occurrence of the G-57T point mutation in the *bla*_OXA-61_ promoter region and an ETP-R phenotype could be established, as these genes were present in both susceptible and resistant isolates.

## DISCUSSION

We sampled 51 flocks of different chicken fattening types on 14 organic farms in Germany to assess the prevalence and antimicrobial resistance of thermotolerant *Campylobacte*r at different stages toward the end of the fattening period. The first samples were collected toward the end of the indoor rearing phase, approximately 1 month after hatching, to address the prevalence and antimicrobial resistance of *Campylobacter* before first outdoor access. Thereafter, samplings were conducted 2 weeks after the onset of outdoor access and at the end of the fattening period for comparative analysis.

### High prevalence of *Campylobacter* in organic meat chicken flocks

In our study, 66.7–69.2 % of samples from male layers and dual-purpose cockerels and 90.0% from slow-growing broilers were already positive for *Campylobacter* at 25–41 days of life before the onset of outdoor access. A similar prevalence (71.2%) was assessed in French free-range slow-growing broiler flocks at the end of the indoor period, where warmer season and insufficient biosecurity measures were significant risk factors for *Campylobacter* colonization before outdoor access was granted (41). In our study, only one flock at farm 12 remained negative for *Campylobacter* throughout the entire fattening period from January to April. This farm was the only one complying with the necessary steps for a functional hygiene lock, in comparison to other study farms. It was also one of two farms that regularly showed first *Campylobacter*-positive samples at S4 instead of S3.

Overall, 98% flock prevalence with 92.3–100% of positive pool samples at the end of the fattening period was observed. In contrast, recent findings from Germany demonstrated lower prevalence in pooled feces (67.1%) from conventional broiler flocks at the end of the fattening period (42) and in broiler cecal samples from the German National Zoonosis Monitoring at slaughterhouses (40.3%) (43). These findings are in line with previous observations of higher *Campylobacter* prevalence in organic compared to conventional broilers in several European countries and the United States (12, 15–18). However, a more recent American study showed higher initial prevalence in conventional broiler flocks upon arrival at the slaughterhouse (24).

The risk of introducing *Campylobacter* to the slaughterhouse increases with a higher prevalence at slaughter age. Carcasses from both organic and comparable extensive free-range flocks were more frequently contaminated with *Campylobacter* post-chilling compared to conventional flocks in studies from Denmark and France (18, 44). Results from the German National Zoonosis Monitoring for fresh chicken retail meat demonstrated similar detection rates, with only a slightly higher prevalence in organic products (46.5 vs. 41.0%) (43). Studies from the United States have reported inconsistent findings regarding *Campylobacter* prevalence for retail meat: some found no significant difference between organic and conventional products (45–48), others reported a higher prevalence in organic meat (49), and a few observed variations depending on the organic brand (50). These discrepancies suggest that factors beyond the overall husbandry system, such as geographical differences, associated producers, or slaughterhouse hygiene, may play a significant role.

Higher slaughter age and access to pasture, with exposure to soil, surface water, and wildlife as vectors for *Campylobacter*, are often discussed as causes for higher prevalence in organic and free-range meat chickens. As access to the outdoors is both linked to favorable seasonal conditions and longer rearing periods, the separation of individual effects of age and outdoor exposure remains challenging in commercially reared flocks. Flocks in our study were slaughtered at 61–138 days, depending on fattening type, while conventional flocks are usually slaughtered between 29 and 42 days of life. Higher age has been associated with increased *Campylobacter* prevalence in some risk factor studies carried out in broilers (51–53). However, this association is not consistently found in all studies (14, 54–56). Notably, most studies on conventional broilers focus on a narrow age range below the slaughter ages typical for organic meat chickens and do not necessarily include slow-growing chicken genotypes at all. Interestingly, *Campylobacter* prevalence did not differ between husbandry systems in poultry reared to a higher age than meat chickens, such as turkeys or laying hens, despite outdoor access only being granted to organic birds (17, 57).

### Rates of antimicrobial resistance in organic meat chicken flocks

Resistance to ciprofloxacin was high to extremely high in both *C. jejuni* and *C. coli*, depending on fattening type at the end of the fattening period (S5). As the vast majority of chickens slaughtered in Germany are fattened in conventional farms, bi-annual resistance results from AMR surveillance programs primarily represent the conventional meat chicken sector. In comparison to *C. jejuni* isolates from German broiler cecal content at slaughter in 2024 (77.1 %), isolates from organic slow-growing broilers (37.0%) and male layer hybrids (59.1%) showed lower resistance rates. The share of CIP-R isolates in dual-purpose cockerels (83.3%) was similarly high as in conventional birds at slaughter. Similar differences could be observed for *C. coli*, where resistance to CIP was slightly lower in isolates from organic slow-growing broilers (64.3%) and male layer hybrids (75.0%) compared to *C. coli* from German broiler cecal content at slaughter (90.7%). Again, the share of CIP-R isolates in dual-purpose cockerels (90.0%) was similarly high in comparison (7). As the total number of *C. coli* isolates recovered for each fattening and sampling time point was low, these results must be interpreted with caution.

Our findings partly confirm a lower resistance rate in organic meat chicken compared to conventional as reported in previous studies. However, in our study, CIP resistance in *C. jejuni* from German organic meat chicken farms was far more prevalent compared to broiler flocks from the United States both before (0% organic, 45.5% conventional) and after (3.7% organic, 8.0% conventional) the ban of fluoroquinolones for the poultry sector in 2005 (17, 24). The prevalence of fluoroquinolone-resistant isolates in our study was also notably higher in comparison to CIP-R *C. jejuni* from Swedish broilers (0% organic, 22.5% conventional) (15) and enrofloxacin-resistant isolates from Danish broilers (0/21 *Campylobacter* isolates organic, 1/12 *Campylobacter* isolates conventional) (16).

In both Denmark and Sweden, use of fluoroquinolones and overall use of antimicrobials in livestock animals is lower compared to Germany (58), and fluoroquinolones are not used at all in Swedish poultry production (15). In Germany, the use of fluoroquinolones for livestock animals is not prohibited but restricted to be used based on antimicrobial resistance testing. A continued use of fluoroquinolones in livestock/poultry farms could possibly contribute to an increased selection pressure for the overall *Campylobacter* population adapted to meat chicken. The use of fluoroquinolone in broiler flocks was previously shown to rapidly select for a variety of resistant strains (59) and is an important risk factor for the detection of CIP-R *Campylobacter* in poultry husbandry (60). Nevertheless, CIP-R *Campylobacter* can also be detected in flocks not previously treated with fluoroquinolones (59, 60). Fluoroquinolone-resistant isolates could be introduced into flocks from the farm environment, as improved hygiene was shown to mitigate the probability of detection on poultry farms, whereas potential contact with wild birds increased it (60). Introduction of strains from the outside seems plausible for slow-growing broilers in our study, as the odds of detecting CIP-R isolates significantly increased with age and outdoor access at S4 compared to S3. However, this association was not visible in other fattening types and toward the end of the fattening period (S5) for slow-growing broilers.

Fluoroquinolone resistance was widespread among different MLST of *C. jejuni* in our study, including clonal complexes ST21-CC and ST353-CC. These clonal complexes have been previously identified as most common among human and animal CIP-R isolates from Central to Eastern Europe, with ST50 being the most common MLST overall (61). In contrast, ST45 was the most prevalent MLST for both organic and conventional broiler flocks in Sweden, and all isolates of this ST were susceptible (15). Some susceptible ST45 *C. jejuni* were isolated from two farms (slow-growing broilers and male layer hybrids) in our study as well.

All but one CIP-R *Campylobacter* isolates in our study carried the *gyrA* p. T86I mutation. This mutation is most commonly observed in fluoroquinolone-resistant *Campylobacter* independent of origin (15, 61, 62). In the absence of antibiotic selection pressure, some *C. jejuni* strains with the *gyrA* p. T86I mutation were shown to colonize and persist in chicken at similar rates as fluoroquinolone-susceptible strains, and were able to outcompete them when co-inoculated (63). These findings help to explain the persistence of isolates carrying this mutation within organic meat chicken husbandry, even though resistance itself poses no fitness advantage given the lack of antimicrobial treatments.

Similar results, albeit at a slightly lower prevalence, were observed for tetracycline resistance within our study compared to results from conventional German broiler production. For *C. jejuni*, resistance to TET was slightly lower in isolates from organic slow-growing broilers (41.3%) and male layer hybrids (59.1%) compared to isolates from broiler cecal content at slaughter in Germany (71.2%). The share of TET-R isolates in dual-purpose cockerels (75.9%) was similarly high in comparison (7). In *C. coli*, TET resistance was more common in isolates from organic slow-growing broilers and dual-purpose cockerels (both 100%) than from conventional broiler isolates from the national AMR monitoring (66.7%), but small isolate numbers of multiple clones from the same farms could bias this result. High levels of tetracycline resistance among isolates from organic broilers (60.0% organic vs 84.4% conventional) have been noted in the United States (17), with TET resistance even being slightly more prevalent in organic isolates (81.6% organic vs 65.3% conventional) in a more recent study (24). For Sweden, TET-R *C. jejuni* were detected at a low but similar rate in organic (3.8%) and conventional (5.6%) broilers (15). Tetracycline is a commonly used antimicrobial in the veterinary sector licensed for most livestock species and part of first-line antimicrobial therapy. Consequently, tetracycline resistance is widespread among *Campylobacter* in different livestock species (7, 64).

Our study demonstrated that ETP-R isolates are also prevalent among the organic meat chicken sector, mainly in *C. coli*. Ertapenem is a carbapenem that has been introduced to the harmonized European AMR surveillance testing panel in 2020 following Commission Implementing Decision (EU) 2020/1729. Carbapenems are not allowed for use in livestock animals in Europe, but were included into monitoring programs on account of case reports of emerging human infections with carbapenem non-susceptible *C. jejuni* (65). The moderate to high occurrence of ETP-R *Campylobacter* in livestock in the European AMR surveillance programs came as a surprise (7, 64). Wild-type MIC distributions, resistance mechanisms as well as the suitability of the current ECOFF of >0.5 mg/L and suitability of ETP within the harmonized testing panel are currently under review by the European Reference Laboratory for Antimicrobial Resistance (64). In the future, the ECOFF for ETP in European AMR surveillance programs might be increased to better reflect the wild-type *Campylobacter* population, consequently requiring a reassessment of ETP-R resistance levels in different populations, including this study. The exact mechanisms of carbapenem resistance in the absence of carbapenemases in *Campylobacter* are a subject of ongoing scientific debate, with synergistic effects of mutations in the genes *porA* (modification of the major outer membrane protein), *cmeABC* (modification of the efflux pump system), and an overexpression of *bla*_OXA-61_ beta-lactamases being discussed as a genetic basis (66–68). In our data set, genes of the blaOXA-61 family with the point mutation G-57T in the promoter region regulating gene expression occurred in both ETP-R- and ETP-susceptible isolates alike, thus showing no clear association. This is in line with a recent study demonstrating that for an ETP-R phenotype, the presence of *porA* mutations was more important than an overexpression of blaOXA-61, whereas the presence of both a *porA* mutation and a G → T transversion in blaOXA-61 was linked to an MIC increase of meropenem (68).

Resistance to chloramphenicol, gentamicin, and erythromycin was not observed in *Campylobacter* isolates from organic meat chickens. This is consistent with results from AMR surveillance in German conventional broilers, where only erythromycin resistance is observed rarely in *C. jejuni* (0.6%) and moderately frequently in *C. coli* (18.5%) (7). As described in other studies (62, 69, 70), the A103V point mutation of the 50S ribosomal protein L22 was not associated with macrolide resistance and should be deleted as a resistance marker from the AMRFinder database.

### Risk factors for ciprofloxacin and tetracycline resistance in *C. jejuni* over the course of fattening

The association between the occurrence of CIP- or TET-resistant *C. jejuni* and season, sampling time point, antimicrobial treatment, and the interaction between sampling time point and fattening type were assessed through generalized linear mixed models. Additionally, whole-genome sequencing of representative isolates from each sample was used to investigate differences in *C. jejuni* genotype distribution between variables significantly associated with the occurrence of resistant isolates. As the resistance profile was mostly consistent among isolates of a certain sequence type, variance could also be interpreted as circulation of different sequence types within each flock. However, a combination of CIP and TET resistance is also a common resistance pattern shared by several *C. jejuni* genotypes.

Statistical analysis revealed similarity in resistance between isolates from the same flock and farm. This clustering effect was confirmed through phylogenetic analysis, as closely related isolates were detected multiple times in different subsequent flocks of a farm. This could reflect potential reservoirs for certain strains in the farm environment. Such reservoirs could include other livestock species (71, 72), especially cattle in the case of meat chickens (73), or contaminated surface water, equipment, and soil in the environment itself (40, 71, 74). Other livestock species like turkeys, ducks, geese, pigs, goats, and (dairy) cattle were kept in addition to meat chickens on some farms but were not sampled and examined within our study. A potential overlap of resistance patterns and genotypes between different livestock on farms was therefore not assessed. Regarding contaminated surface waters and soil, the outdoor run of organic chickens might serve as a reservoir for *Campylobacter* strains from previous flocks. Recent experimental studies under laboratory conditions indicate that *Campylobacter* in a viable-non-culturable state could possibly persist in soil and water in the environment for several weeks given favorable climate conditions (high humidity, low UV radiation, and mild temperatures) (75). Nevertheless, no clear transmission pathways of *Campylobacter* between environmental reservoirs and flocks have been established yet (40, 71).

Season was significantly associated with the occurrence of resistant isolates, with higher odds in autumn for CIP-R and higher odds in warmer seasons compared to winter for TET-R. Concurrently, a seasonal variation and temporal shift in strain circulation could also be observed within flocks and between consecutive flocks on several farms. A seasonal variation of circulating *Campylobacter* genotypes has been described before for isolates from human cases, poultry, milk, and water (76–78). Furthermore, subsequent flocks of the same farm reared and fattened during the summer did not necessarily share any *Campylobacter* genotypes between 2023 and 2024 in our study. The reason for seasonal variation in *Campylobacter* prevalence in broilers is often attributed to a confounding seasonal influence on the activity of potential vectors such as flies (79–81). A seasonal variation in *Campylobacter* subtypes could indicate a shift in the importance of different transmission routes into meat chicken farms, especially during colder seasons with less vector activity.

The interaction between fattening type and sampling time point was also significant. Despite flocks of all fattening types being of the same age range at S3, the occurrence of CIP- and TET-resistant strains differed between fattening types. This can be related to certain CIP-TET resistance-associated MLST being repeatedly isolated at S3 from certain slow-growing broiler or dual-purpose cockerel farms, like ST6175 from all four flocks on farm 1 or ST9897 from the majority of flocks from farms 6 and 7. It is unclear whether this represents an actual influence of the fattening type in question or a common source of these strains in the environment that was simply absent from other farms based on differences in geography, feed supply, and management. As only a few farms per fattening type were sampled, rare events on singular farms may disproportionately affect statistical significance. The wide confidence intervals show a high degree of statistical uncertainty regarding the extent of these differences. Otherwise, it could be hypothesized that different *Campylobacter* genotypes preferably colonize certain chicken genotypes based on differences in the host immune system, morphology, and gut microbiome. Experimental studies indicated that chicken genotype itself might influence the general ability of certain *Campylobacter* strains to colonize the host gut, leading to reduced bacterial loads (82, 83). However, the extent of genetic control for *Campylobacter* in commercial chicken genotypes following natural exposure is likely limited (84).

The fattening types also exhibited different patterns in the occurrence of CIP- and TET-resistant *C. jejuni* over time at S4 and S5. The predominant strains in organic and free-range broilers were previously shown to change throughout the fattening period based on PFGE-typing (13, 85). As the resistance profile was consistent for isolates of each PFGE-type, a difference in *Campylobacter* genotypes could explain the differences in AMR between free-range and organic broiler flocks (13). Similarly, the increase and decrease of tetracycline resistance within an organic broiler flock was based on the shift of predominant-resistant or -susceptible strains over time (85). It is unclear whether these changes were really dependent on the fattening type itself. These shifts over the fattening period might rather follow the changing composition of the chicken’s gut microbiome over time, which is influenced by the combination of age, host genotype, housing conditions, veterinary treatments, changing feed throughout the fattening period, and, again, season (86).

### Limitations

The choice of organic farms available to the study was limited by personnel restraints, as well as the defined genotypes of chicken. To model a potential effect of the fattening type in commercial farming settings, only flocks of certain genotypes were included to exclude additional genetic variety as best as possible. Several flocks per farm were, therefore, examined to account for farm effects. The farms needed to be in a region accessible to the project partners at the University of Kassel, which is why most farms were located in Western to Central Germany. However, this covered the regions with the highest density of poultry and other livestock in Germany.

The wide confidence intervals in pairwise comparison demonstrate a high degree of statistical uncertainty in the results, which is likely related to small sample sizes per subgroup. While the sequential Bonferroni correction as a more conservative method to assess significance in multiple comparisons was used, the significant results should be interpreted as a potential trend to be verified in future studies with larger sample sizes. Future studies should also include standardized biosecurity scores fit to assess free-range poultry systems, such as Biocheck.UGent (87). Biosecurity and management factors represent important determinants of farm hygiene and therefore transmission pathways that were likely not fully captured by the farm-specific random factor in our study design. Similarly, the presence of other livestock on farm likely represents an important reservoir for *Campylobacter*, especially when sharing pastures.

Due to conducting a longitudinal observational study, the variables age and outdoor access, as well as fattening type and more pronounced age differences at sampling time points S4 and S5 were closely correlated. Potential differences between the fattening types at S4 and S5 could therefore be based on the resulting age difference of the birds and resulting differences in maturity and development of the gut microbiome instead of genetic effects. Interventional studies with controlled rearing conditions and control groups per fattening type are needed to examine the single effect of age, outdoor access, and chicken genotype. However, in the commercial settings represented by our study, these factors will always be interwoven, as the lower feed conversion rates of male laying hybrids and dual-purpose cockerels require longer fattening periods and consequently longer periods of outdoor access in comparison to slow-growing fattening hybrids.

The genotypic characterization of isolates consisted of a qualitative analysis of MLST present in the population of each flock and sampling time point. Due to four *Campylobacter* colonies per sample being speciated and one representative for each resistance pattern and species from each sample being chosen for WGS, the proportions of each *Campylobacter* genotype cannot be assessed with our methods. Nevertheless, the variety of the population structure as a whole and the presence of certain genotypes common to the conventional sector was demonstrated.

### Conclusion

Our study confirmed an overall high prevalence of 98% of *Campylobacter* in organic meat chicken flocks at the end of the fattening period. Strikingly, even prior to outdoor access, a majority of the flocks were positive for *Campylobacter*. A high risk of transmission into slaughterhouses and the subsequent processing line cannot be dismissed. This underlines the importance of improved slaughter hygiene and consumer education on kitchen hygiene regardless of production labels.

Despite low antimicrobial use in all farms, *Campylobacter* isolates showed high rates of ciprofloxacin and tetracycline resistance in all three fattening types. However, in contrast to conventional husbandry, all isolates were sensitive to macrolides. Ertapenem resistance was observed in *C. jejuni* and *C. coli* from some farms. Here, it will be of future interest to establish the driving factors for carbapenem resistance development in chicken, since carbapenems are not used for livestock. Changes in the occurrence of ciprofloxacin- and tetracycline-resistant *C. jejuni* isolates were significantly associated with season and the combination of sampling time point and fattening type. Whole-genome sequencing of representative isolates reflected an individual circulation of resistant genotypes both within a flock and across subsequent flocks of the same farm. Clonal complexes common to conventional broiler production, like ST21-CC, ST353-CC, and ST828-CC, could be identified in flocks from all fattening types. In combination with resistance mechanisms that do not necessarily induce fitness losses for *Campylobacter* colonization, like *gyrA* p. T86I and the *tet(O*) gene, this might explain high resistance rates to ciprofloxacin and tetracycline similar to conventional broiler production regardless of antimicrobial use.

Our study highlights the widespread occurrence of resistant *Campylobacter* even within organic husbandry systems. This emphasizes the need for future research regarding the transmission pathways and reservoirs of *Campylobacter* for organic chicken flocks, incorporating data on internal and external biosecurity.

## Supplementary Material

Reviewer comments

## Data Availability

Assembled genome sequences and trimmed reads were provided at the National Center for Biotechnology Information (NCBI) under BioProject PRJNA1338716.

## AFTER EPUB

*[This article was published on 4 September 2026 with formatting errors. The errors were corrected in the current version, posted on 28 September 2026.]*

## SUPPLEMENTAL MATERIAL

File S1 spectrum.01012-26-s0001.pdf Additional farm information on livestock and biosecurity data.

File S2 spectrum.01012-26-s0002.pdf Additional information for GLMM analysis.

File S3 spectrum.01012-26-s0003.pdf Minimum spanning trees of cgMLST based clusters for *C. jejuni* and *C. coli*.

Tables S1 to S4 spectrum.01012-26-s0004.xlsx Isolate level data on MIC and ARG, Aquamis QC, Aquamis tresholds and accession numbers.

OPEN PEER REVIEW reviewer-comments.pdf An accounting of the reviewer comments and feedback.

ASM does not own the copyrights to Supplemental Material that may be linked to, or accessed through, an article. The authors have granted ASM a non-exclusive, world-wide license to publish the Supplemental Material files. Please contact the corresponding author directly for reuse.
