# Supplementary material for: Longitudinal study of prevalence and antimicrobial resistance of thermotolerant Campylobacter spp. in German organic meat chicken farms
Source: Microbiol Spectr. 2026 Sep 4;14(10):e01012-26. doi: 10.1128/spectrum.01012-26 (PMC13689127; doi:10.1128/spectrum.01012-26)
Supplement: Supplementary file 1 [file reviewer-comments.pdf]

ASM | Microbiology Spectrum

Longitudinal study of prevalence and antimicrobial resistance of thermotolerant Campylobacter spp. in German organic meat chicken farms

Anna Korves-Wilm, Kerstin Stingl, Margret Krieger, Mirjam Grobbel, and Bernd-Alois Tenhagen

Corresponding Author(s): Anna Korves-Wilm, Bundesinstitut fur Risikobewertung

Review Timeline:

Submission Date:	March 24, 2026
Editorial Decision:	May 26, 2026
Revision Received:	July 24, 2026
Accepted:	August 5, 2026

Editor: Orhan Sahin

Reviewer(s): Disclosure of reviewer identity is with reference to reviewer comments included in decision letter(s). The following individuals involved in review of your submission have agreed to reveal their identity: Palmy Jesudhasan (Reviewer #3); Hosny El-Adawy (Reviewer #4)

Transaction Report:

(Note: With the exception of the correction of typographical or spelling errors that could be a source of ambiguity, letters and reports are not edited. The original formatting of letters and referee reports may not be reflected in this compilation.)

DOI: https://doi.org/10.1128/spectrum.01012-26

Re: Spectrum01012-26 (Longitudinal study of prevalence and antimicrobial resistance of thermotolerant Campylobacter spp. in German organic meat chicken farms)

Dear Dr. Anna Maria Korves-Wilm:

Thank you for the privilege of reviewing your work. Below you will find my comments, instructions from the Spectrum editorial office, and the reviewer comments.

Please return the manuscript within 60 days; if you cannot complete the modification within this time period, please contact me. If you do not wish to modify the manuscript and prefer to submit it to another journal, notify me immediately so that the manuscript may be formally withdrawn from consideration by Spectrum.

Revision Guidelines
To submit your modified manuscript, log into the submission site at https://spectrum.msubmit.net/cgi-bin/main.plex. Go to Author Tasks and click the appropriate manuscript title to begin. The information you entered when you first submitted the paper will be displayed; update this as necessary. Note the following requirements:

• Upload point-by-point responses to the issues raised by the reviewers in a file named "Response to Reviewers," NOT in your cover letter.
• Upload a compare copy of the manuscript (without figures) as a "Marked-Up Manuscript" file.
• Upload a clean .DOC/.DOCX version of the revised manuscript and remove the previous version.
• Each figure must be uploaded as a separate, editable, high-resolution file (TIFF or EPS preferred), and any multipanel figures must be assembled into one file.
• Any supplemental material intended for posting by ASM should be uploaded with their legends separate from the main manuscript. You can combine all supplemental material into one file (preferred) or split it into a maximum of 10 files with all associated legends included.

For complete guidelines on revision requirements, see our Submission and Review Process webpage. Submission of a paper that does not conform to guidelines may delay acceptance of your manuscript.

Data availability: ASM policy requires that data be available to the public upon online posting of the article, so please verify all links to sequence records, if present, and make sure that each number retrieves the full record of the data. If a new accession number is not linked or a link is broken, provide Spectrum production staff with the correct URL for the record. If the accession numbers for new data are not publicly accessible before the expected online posting of the article, publication may be delayed; please contact production staff (Spectrum@asmusa.org) immediately with the expected release date.

Publication Fees: For information on publication fees and which article types are subject to charges, visit our website. If your manuscript is accepted for publication and any fees apply, you will be contacted separately about payment during the production process; please follow the instructions in that e-mail. Arrangements for payment must be made before your article is published.

ASM Membership: Corresponding authors may join or renew ASM membership to obtain discounts on publication fees. Need to upgrade your membership level? Please contact Customer Service at Service@asmusa.org.

The ASM Journals program strives for constant improvement in our submission and publication process. Please tell us how we can improve your experience by taking this quick Author Survey.

Thank you for submitting your paper to Spectrum.

Sincerely,
Orhan Sahin
Editor
Microbiology Spectrum

Reviewer #1 (Public repository details (Required)):

Provide all Campylobacter isolates uploaded to PubMLST (PubMLST ID), as well as SRA.

Reviewer #1 (Comments for the Author):

This manuscript reports a longitudinal study of Campylobacter prevalence and antimicrobial resistance (AMR) across 14 German certified organic meat chicken farms, spanning three fattening types: slow-growing broilers, male layer hybrids, and dual-purpose cockerels. It encompasses 51 flocks sampled at three key time points between 2023 and 2025.
The study integrates phenotypic AMR testing, whole-genome sequencing (WGS) of representative isolates and generalized linear mixed models (GLMM). The addresses a genuine knowledge gap regarding AMR dynamics in alternative chicken fattening systems that have become increasingly important since Germany's 2022 ban on male chick culling.
The study's principal strengths are its longitudinal design tracking multiple flocks per farm, its novel inclusion of male layer hybrids and dual-purpose cockerels as fattening types, the use of an appropriate hierarchical statistical model, and the striking observation that the majority of flocks were Campylobacter-positive before first outdoor access.
Comments:
1. The authors describe read trimming (Trimmomatic) and species confirmation (MASH) but do not report genome completeness or contamination metrics. Tools such as CheckM2 should be applied to all assemblies. The authors should provide in supplementary data, per-assembly statistics including N50, total assembly length, number of contigs, and completeness/contamination estimates (e.g., CheckM completeness {greater than or equal to}95%, contamination {less than or equal to}5%). Any assemblies failing these thresholds should be excluded and their exclusion noted, with a statement on whether removal affected the study conclusions. This is particularly important to determine if ertapenem resistance was not observed due to contamination, which is quite common.
2. The authors acknowledge that sampling time point reflects both bird age and duration of outdoor access, and that these two variables cannot be separated statistically. This limitation deserves a stronger discussion than currently provided. At the later sampling points (S4 and S5), the three fattening types differ considerably in age: slow-growing broilers are 64 days old at S4, male layer hybrids 71 days, and dual-purpose cockerels 77 days. As a result, any observed differences between fattening types at these time points may reflect bird age rather than, or in addition to, genotype or outdoor access. The authors should report these age differences explicitly in the methods or a supplementary table, and the discussion of fattening type effects on Campylobacter AMR patterns should be acknowledged and discussed.
3. The authors selected one isolate per species per unique resistance profile per pool for whole-genome sequencing. While this approach is practical, it means that common resistance profiles and rare ones each contribute a single sequence regardless of how frequently they actually occurred. A strain found in 80% of isolates from a pool is represented by the same one genome as a strain found only once. As a result, the phylogenetic trees and clonal complex distributions shown in the study reflect the diversity of resistance profiles rather than their true prevalence in the sampled flocks. This is a meaningful limitation when interpreting population structure, and it is currently mentioned only briefly. The authors should include a dedicated limitation paragraph addressing this issue, and statements such as "the majority of C. jejuni belonged to the ST21 clonal complex" should be modified to reflect that this conclusion is based on a sampling strategy that was not designed to capture population proportions.
4. Ertapenem-resistant C. coli was found at strikingly high rates: 69.6% overall over 90% in some subgroups, despite carbapenems being banned from use in livestock, and despite the fact that carbapenem resistance in Campylobacter have been rarely described. If these findings are real this would represent a major public health concern. The authors only queried carbapenem resistance genes using AMRFinder, and this approach has a critical limitation: no carbapenem resistance determinants for Campylobacter are currently defined in the NCBI resistance gene database, meaning AMRFinder is essentially guaranteed to return no hits. It is also important to note that the authors do not report the presence of intrinsic OXA-type beta-lactamases, which are commonly found in Campylobacter and routinely detected by AMRFinder, and which were previously reported in a case of carbapenem resistant Campylobacter associated bacteriemia (PMID: 39316322). Their absence from the results is unexpected and raises questions about whether the AMRFinder analysis was run correctly or in organism-specific mode. To investigate potential genomic correlates of the ETP-R phenotype, the authors should perform a comparative genomic analysis of ETP-R versus ETP-S C. coli isolates, specifically examining efflux pump genes (e.g., RE-cmeABC), beta-lactam-related loci, etc (PMID: 37358443). A GWAS approach would be ideal but it is acknowledged that isolate numbers may not be sufficient for a robust analysis. The discussion should more substantively consider alternative explanations for the ETP-R phenotype, as well as the possibility that the current ECOFF is simply too low and is capturing natural biological variation in wild-type Campylobacter rather than true resistance. The authors should also discuss the striking farm-level clustering: farm 4 had 80% of its C. coli testing ETP-R, and farm 13 had 92.9%. These farms stand out clearly from the rest, and the paper does not attempt to explain what, if anything, was different about them.
5. The authors should provide a phenotype-to-genotype comparison for all antimicrobial resistance results reported in the study. For each resistance phenotype, the corresponding genomic determinant(s) detected should be listed, and any discordant cases should be identified and discussed.
6. Farm 8 specializes in finishing birds reared on farms 6 and 9, and was sampled only at S4 and S5 while farms 6 and 9 were sampled at S3 only. The farms are epidemiologically linked, sharing chick batches and demonstrably sharing clonal Campylobacter strains. Yet in the statistical models, Farm 8 is treated as an independent farm with its own random intercept. The authors should clarify how the linked farms were handled statistically, or perform a sensitivity analysis excluding Farm 8 from the GLMM to assess whether findings change substantially.
7. The GLMM includes meteorological season as a fixed effect with significant results reported, but the methods do not define which months correspond to which season. Please modify.
8. There are only four to five farms per fattening type, and thus the pairwise contrasts yields odds ratios with extremely large confidence intervals (Table 3: OR = 41.0, 95% CI 1.3-1262.4 for TET resistance, dual-purpose vs. male layer; Table 4: OR = 3678.6, 95% CI 43.3-312582.6 for TET at S3, dual-purpose vs. male layer). The statistical significance of these estimates should be interpreted with caution. The discussion needs to flag that statistical significance in the context of such imprecision should not

be mistaken for biological certainty. Please update the discussion and limitations.
9. The biosecurity questionnaire data collected should be presented descriptively in a supplementary table, and authors should acknowle that the inability to include biosecurity data as a covariate is a major limitation of the study design. Please update limitations section.
10. Line 403: "Isolates not assigned to ST829-CC" typo for "ST828-CC."?
11. Lines 540-541 state that "all CIP-R Campylobacter isolates carried the gyrA T86I mutation. However, later in the manuscript it is reported that one CIP-R isolate with a MIC of 1 mg/L where gyrA T86I was not detected. Please harmonize.
12. The authors state that MLST and AMR screening were performed using the BakCharak pipeline (gitlab.com/bfr_bioinformatics/bakcharak), but the description provided is insufficient for reproducibility. Please clarify: 1. the version of BakCharak. 2. since the MLST module within BakCharak relies on SISTR it is unclear which tool and allele database were used for Campylobacter MLST. 3. Confirm AMRFinder was used using the organism specific mode (--organism Campylobacter); also confirm AMRFinder database version used.

Reviewer #3 (Comments for the Author):

Manuscript entitled "Longitudinal ......farms" is well written. The experiments are well designed and it is an interesting and a timely article.

Reviewer #4 (Comments for the Author):

Reviewer # (Comments for the Author):
The manuscript (control no. Spectrum 01012-26) entitled "Longitudinal study of prevalence and antimicrobial resistance of thermotolerant Campylobacter spp. in German organic meat chicken farms". This study aimed to examine the prevalence and antimicrobial resistance of thermotolerant Campylobacter spp. in fourteen German organic meat chicken farms rearing slow-growing broiler, male layer hybrids or dual-purpose cockerels in a longitudinal study.
The results revealed that at the end of the fattening period, 98 % of all flocks were positive with C. jejuni being the predominant species. Antimicrobial resistance of 405 C. jejuni and 79 C. coli revealed overall high levels of ciprofloxacin and tetracycline resistance but absence of macrolide resistance. Results for flocks within farms showed clustering effects (ICCCIP = 0.447; ICCTET = 0.412). Whole genome sequencing of representative isolates confirmed great overall genetic variety, with farm- or fattening type-dependent dissemination of certain multi-locus sequence types. The majority of C. jejuni belonged to ST21 clonal complex. The majority of C. coli belonged to ST828 clonal complex.
This manuscript is well written, the experiment is well designed and the results is correctly illustrated in different table and informative figures.
General comment:
1. Although this manuscript described an essential research point for a significant foodborne pathogen in organic meat chicken farms, this study did not discuss all epidemiological aspects of the farms and if there is any contact with other animals might be as a source of transmission.
2. This study performed in organic poultry farms and the authors (lines 153-156) showed the antimicrobial treatment of flocks, could you briefly expand this point and show the reason of medication to give the reader have an overview about the previously published data on the same flocks.
3. In the title, the authors give us a quick feeling that the samples were collected from slaughterhouses "German organic meat chicken farms", it will be better if you replace it either with broilers or poultry farms.
4. In methodology section, the author needs to make the study design more concise and don't build on the previous part of the work which previously published elsewhere. It means, the reader needs to read the previous publication to understand the current one.
5. In line 162: please add the method to adjust the microaerophilic condition.
6. In the result section (Lines 276-280), considered as part of methodology section.
7. In spite of the manuscript is well written and informative, there are some grammatic and typing errors in the manuscript. The authors should revise the whole manuscript for these minimal typing mistakes (e.g. lines 17; 20; 29....).
8. In the result section "Resistance determinants and phenotypical resistance in Campylobacter", line 434: the authors used AMRFinder database, please clarify why only this tool used and not others as ResFinder or NCBI, etc......
9. The discussion section is very long and need to be summarized.
10. In Table 1: in the raw of slow growing flock, the total positive was 18 out 20 but in the column of C. jejuni and C. coli were mire than 18. Please clarify.

Reviewer # (Comments for the Author):

The manuscript (control no. Spectrum 01012-26) entitled "Longitudinal study of prevalence and antimicrobial resistance of thermotolerant *Campylobacter* spp. in German organic meat chicken farms". This study aimed to examine the prevalence and antimicrobial resistance of thermotolerant *Campylobacter* spp. in fourteen German organic meat chicken farms rearing slow-growing broiler, male layer hybrids or dual-purpose cockerels in a longitudinal study.

The results revealed that at the end of the fattening period, 98 % of all flocks were positive with *C. jejuni* being the predominant species. Antimicrobial resistance of 405 *C. jejuni* and 79 *C. coli* revealed overall high levels of ciprofloxacin and tetracycline resistance but absence of macrolide resistance. Results for flocks within farms showed clustering effects ($ICC_{CIP} = 0.447$; $ICC_{TET} = 0.412$). Whole genome sequencing of representative isolates confirmed great overall genetic variety, with farm- or fattening type-dependent dissemination of certain multi-locus sequence types. The majority of *C. jejuni* belonged to ST21 clonal complex. The majority of *C. coli* belonged to ST828 clonal complex.

This manuscript is well written, the experiment is well designed and the results is correctly illustrated in different table and informative figures.

General comment:

1. Although this manuscript described an essential research point for a significant foodborne pathogen in organic meat chicken farms, this study did not discuss all epidemiological aspects of the farms and if there is any contact with other animals might be as a source of transmission.
2. This study performed in organic poultry farms and the authors (lines 153-156) showed the antimicrobial treatment of flocks, could you briefly expand this point and show the reason of medication to give the reader have an overview about the previously published data on the same flocks.
3. In the title, the authors give us a quick feeling that the samples were collected from slaughterhouses "German organic meat chicken farms", it will be better if you replace it either with broilers or poultry farms.
4. In methodology section, the author needs to make the study design more concise and don't build on the previous part of the work which previously published elsewhere. It means, the reader needs to read the previous publication to understand the current one.
5. In line 162: please add the method to adjust the microaerophilic condition.
6. In the result section (Lines 276-280), considered as part of methodology section.
7. In spite of the manuscript is well written and informative, there are some grammatic and typing errors in the manuscript. The authors should revise the whole manuscript for these minimal typing mistakes (e.g. lines 17; 20; 29….).
8. In the result section "Resistance determinants and phenotypical resistance in *Campylobacter*", line 434: the authors used AMRFinder database, please clarify why only this tool used and not others as ResFinder or NCBI, etc……

9. The discussion section is very long and need to be summarized.
10. In Table 1: in the raw of slow growing flock, the total positive was 18 out 20 but in the column of *C. jejuni* and *C. coli* were mire than 18. Please clarify.

Author's response to reviewer's comments on manuscript Spectrum01012-26

Reviewer #1 (Public repository details (Required)):

Provide all Campylobacter isolates uploaded to PubMLST (PubMLST ID), as well as SRA.

All trimmed sequences for this study were uploaded to SRA under bioproject PRJNA1338716.
All genome assemblies were submitted to the genome collection of PubMLST.
The accessions numbers were provided in **Supplementary Material S2 Table 4**.
The corresponding records are currently under embargo and are not yet publicly searchable. The records are scheduled for public release upon publication of the article. We have verified that the accession numbers are correct and will ensure that they are publicly accessible no later than the online publication date.

Reviewer #1 (Comments for the Author):

This manuscript reports a longitudinal study of Campylobacter prevalence and antimicrobial resistance (AMR) across 14 German certified organic meat chicken farms, spanning three fattening types: slow-growing broilers, male layer hybrids, and dual-purpose cockerels. It encompasses 51 flocks sampled at three key time points between 2023 and 2025.
The study integrates phenotypic AMR testing, whole-genome sequencing (WGS) of representative isolates and generalized linear mixed models (GLMM). The addresses a genuine knowledge gap regarding AMR dynamics in alternative chicken fattening systems that have become increasingly important since Germany's 2022 ban on male chick culling.

The study's principal strengths are its longitudinal design tracking multiple flocks per farm, its novel inclusion of male layer hybrids and dual-purpose cockerels as fattening types, the use of an appropriate hierarchical statistical model, and the striking observation that the majority of flocks were Campylobacter-positive before first outdoor access.

We thank reviewer #1 for their thorough evaluation of the manuscript, provided comments and critical questions, which have been very helpful in improving the manuscript. We have addressed all recommendations in the revised version as listed below.

Comments:
1. The authors describe read trimming (Trimmomatic) and species confirmation (MASH) but do not report genome completeness or contamination metrics. Tools such as CheckM2 should be applied to all assemblies. The authors should provide in supplementary data, per-assembly statistics including N50, total assembly length, number of contigs, and completeness/contamination estimates (e.g., CheckM completeness {greater than or equal to}95%, contamination {less than or equal to}5%). Any assemblies failing these thresholds should be excluded and their exclusion noted, with a statement on whether removal affected the study conclusions. This is particularly important to determine if ertapenem resistance was not observed due to contamination, which is quite common.

We have now provided the assembly QC parameters in **Supplementary Material S2 Table 2**. These were generated within initial analyses through AQUAMIS version 1.4.2., and only isolates complying with all quality parameters and thus passing the AQUAMIS quality check were included into our study from the beginning. The *Campylobacter* specific thresholds are also provided within **Supplementary Material S2 Table 3**.

Genome completeness and contamination estimates were generated through QUAST (5.2.0, utilizes BUSCO) and Confindr (version 0.7.4) instead of CheckM2. For *Campylobacter*, a taxon-specific and validated threshold of 79% single copy orthologs (instead of 95%) was used. Please see Deneke et al (2021) (https://doi.org/10.3390/genes12050644) for an in-depth explanation of all utilized applications and thresholds within AQUAMIS.
There was no interspecies contamination flagged for any isolate. Intraspecies contamination was flagged for two isolates (each with 2 contaminating SNVs, 5.63 – 5.76% contamination), but were within quality control thresholds of AQUAMIS (≤ 2 contaminating intra-species SNV). Their inclusion into the study for profiling of MLST and resistance determinants were thus tolerated. As cluster analysis was based on cgMLST allele distance and not on SNP differences, their inclusion in this part of the analysis may also be tolerated.

In summary, ertapenem resistance was not observed due to any contamination or misspecification. The results and discussion are therefore not affected.

2. The authors acknowledge that sampling time point reflects both bird age and duration of outdoor access, and that these two variables cannot be separated statistically. This limitation deserves a stronger discussion than currently provided. At the later sampling points (S4 and S5), the three fattening types differ considerably in age: slow-growing broilers are 64 days old at S4, male layer hybrids 71 days, and dual-purpose cockerels 77 days. As a result, any observed differences between fattening types at these time points may reflect bird age rather than, or in addition to, genotype or outdoor access. The authors should report these age differences explicitly in the methods or a supplementary table, and the discussion of fattening type effects on Campylobacter AMR patterns should be acknowledged and discussed.

We agree that larger differences in age at sampling time points S4 and S5 may pose additional confounding effects when examining differences between fattening types. This has been mentioned in the original manuscript (see line 640-643), but has now been expressed more clearly (see lines 676-680 revised manuscript). Additionally, the correlation of age – outdoor access – fattening type in commercial settings received a subsection in the new limitations section (see lines 700-709 revised manuscript).

As requested, the age differences were addressed more prominently in the methods sections with their own subsection and table compiling all age ranges previously noted in text (lines 139-147 original manuscript; now Table 1 and lines 148-150 in revised manuscript).

3. The authors selected one isolate per species per unique resistance profile per pool for whole-genome sequencing. While this approach is practical, it means that common resistance profiles and rare ones each contribute a single sequence regardless of how frequently they actually occurred. A strain found in 80% of isolates from a pool is represented by the same one genome as a strain found only once. As a result, the phylogenetic trees and clonal complex distributions shown in the study reflect the diversity of resistance profiles rather than their true prevalence in the sampled flocks. This is a meaningful limitation when interpreting population structure, and it is currently mentioned only briefly. The authors should include a dedicated limitation paragraph addressing this issue, and statements such as "the majority of C. jejuni belonged to the ST21 clonal complex" should be modified to reflect that this conclusion is based on a sampling strategy that was not designed to capture population proportions.

Reviewer #1 is correct in pointing this out. We apologize for any misleading phrasing, as we did not mean to invoke otherwise. Corresponding statements in the abstract and results section were

rephrased (see lines 37-39, 387, 394 and 402 in revised manuscript). A dedicated subsection section was added to the limitations in lines 710-715 of the revised manuscript.

4. Ertapenem-resistant C. coli was found at strikingly high rates: 69.6% overall over 90% in some subgroups, despite carbapenems being banned from use in livestock, and despite the fact that carbapenem resistance in Campylobacter have been rarely described. If these findings are real this would represent a major public health concern. The authors only queried carbapenem resistance genes using AMRFinder, and this approach has a critical limitation: no carbapenem resistance determinants for Campylobacter are currently defined in the NCBI resistance gene database, meaning AMRFinder is essentially guaranteed to return no hits.
It is also important to note that the authors do not report the presence of intrinsic OXA-type beta-lactamases, which are commonly found in Campylobacter and routinely detected by AMRFinder, and which were previously reported in a case of carbapenem resistant Campylobacter associated bacteriemia (PMID: 39316322). Their absence from the results is unexpected and raises questions about whether the AMRFinder analysis was run correctly or in organism-specific mode.
To investigate potential genomic correlates of the ETP-R phenotype, the authors should perform a comparative genomic analysis of ETP-R versus ETP-S C. coli isolates, specifically examining efflux pump genes (e.g., RE-cmeABC), beta-lactam-related loci, etc (PMID: 37358443). A GWAS approach would be ideal but it is acknowledged that isolate numbers may not be sufficient for a robust analysis. The discussion should more substantively consider alternative explanations for the ETP-R phenotype, as well as the possibility that the current ECOFF is simply too low and is capturing natural biological variation in wild-type Campylobacter rather than true resistance. The authors should also discuss the striking farm-level clustering: farm 4 had 80% of its C. coli testing ETP-R, and farm 13 had 92.9%. These farms stand out clearly from the rest, and the paper does not attempt to explain what, if anything, was different about them.

All analysis were run correctly. The database versions were added to the methods section (see comment 12 for detailed information).
Please note that we did report on the presence of intrinsic OXA-type beta-lactamases (61 and 184 family) in summarized form in the results section (lines 412-416 of the original manuscript, lines 436-440 revised manuscript) and Figure 5. For each isolate, the phenotype and detected resistance determinants is also included in **Supplementary Material S2 Table 1**. The presence of OXA-type beta-lactamases was not entirely missing from the results and could be traced back to isolate level with the material provided. However, no clear association was found between ETP-R phenotype and presence of OXA-type beta-lactamases. We have clarified this in the results section (lines 455-458 revised manuscript).
In the discussion, the questioned suitability of the current ECOFF, possibility of natural wild type MIC distribution and current review of those factors by the EURL AR was mentioned and cited in lines 567-569 of the original manuscript. We have made the implications for our study clearer and added a discussion of relevant gene modifications (*porA* and *cmeABC*) besides blaOXA-61 p. G-57T (lines 593-606 revised manuscript). Given the focus and aims of our study, conducting a more in-depth genomic analysis of *porA* and *cmeABC* mutations across the isolates is out of scope, especially regarding the manuscript's length, which was already criticized by reviewer #4.

There were no defining characteristics shared by both farm 4 and farm 13 regarding the fattening type used or other livestock reared on farm that made them stand out from other farms. ETP-R isolates from these farms were of different MLST, thus not indicating a reservoir shared by both farms. Based on all data available to us, we could only speculate and thus resorted to describing the observed clusters.

5. The authors should provide a phenotype-to-genotype comparison for all antimicrobial resistance results reported in the study. For each resistance phenotype, the corresponding

genomic determinant(s) detected should be listed, and any discordant cases should be identified and discussed.

We fully agree that such data need to be published to improve databases in the long term. Accordingly, we had provided all phenotype and detected resistant determinants within the **Supplementary Material S2 Table 1** at isolate level and highlighted any discrepancies between pheno- and genotype in the results section (see lines 411-429 in original manuscript). This included the detection of the A103V point mutation of the 50S ribosomal protein L22 in isolates phenotypically susceptible to erythromycin (lines 417-419 and Figure 5 of the original manuscript, lines 441-4443 revised manuscript), with brief discussion of this discrepancy being known and referring to previous literature (lines 573-575 of the original manuscript, lines 610-612 revised manuscript). Otherwise, the presence of non-functional *tet*(O) genes due to premature stop codons being introduced by point mutations in three isolates phenotypically susceptible to tetracycline was highlighted in the results section (lines 425-428 of the original manuscript, lines 451-453 revised manuscript). Such mutations occasionally occurring did not warrant further discussion in our eyes.
As the focus of our study is on assessing which resistance patterns are found within different types of organic meat chicken at different timepoints, we fear that further expansion on these points in the already detailed discussion is out of scope for this publication. We have highlighted the availability of these data in **Supplementary Material S2** in lines 434-435 in the revised manuscript.

Please note that after including an additional analysis through ResFinder, Figure 5 has been renewed to include detection of the tet(32/O/32) gene variant (also see lines 449-451 revised manuscript). **Supplementary Material S2 Table 1** has been updated to include such discrepancies between databases.

6. Farm 8 specializes in finishing birds reared on farms 6 and 9, and was sampled only at S4 and S5 while farms 6 and 9 were sampled at S3 only. The farms are epidemiologically linked, sharing chick batches and demonstrably sharing clonal Campylobacter strains. Yet in the statistical models, Farm 8 is treated as an independent farm with its own random intercept. The authors should clarify how the linked farms were handled statistically, or perform a sensitivity analysis excluding Farm 8 from the GLMM to assess whether findings change substantially.

Reviewer #1 is correct in pointing this out and we apologize for not explaining this in the methods section. This was now added in lines 287-291 of the revised manuscript.
We assumed an independent random intercept for farm 8 only at timepoints S4 and S5, after the birds arrived at their new location. For S3, isolates of the corresponding flocks were coded to belong to a respective dummy flock on farm 6 or 9. The metadata coding for each isolate can be traced back in **Supplementary Material S2 Table 1** via the comment section. This was a practical approach to include a random intercept for the farm and environment where the birds were actually located at the time the sample was taken. We acknowledge that including interconnected farms are not optimal, but there were no other available farms rearing the defined genotype for dual-purpose cockerels.
Excluding farm 8 from the analysis does not substantially change the central findings. We have attached these additional GLMM outputs to the end of this response for comparison.
While the model is not perfect, we hope reviewer #1 finds it sufficient to examine the most important factors at hand at an appropriate level of complexity.

7. The GLMM includes meteorological season as a fixed effect with significant results reported, but the methods do not define which months correspond to which season. Please modify.

We have added an explanation to the methods section (lines 276-278 in the revised manuscript).

8. There are only four to five farms per fattening type, and thus the pairwise contrasts yields odds ratios with extremely large confidence intervals (Table 3: OR = 41.0, 95% CI 1.3-1262.4 for TET resistance, dual-purpose vs. male layer; Table 4: OR = 3678.6, 95% CI 43.3-312582.6 for TET at S3, dual-purpose vs. male layer). The statistical significance of these estimates should be interpreted with caution. The discussion needs to flag that statistical significance in the context of such imprecision should not be mistaken for biological certainty. Please update the discussion and limitations.

We agree and have addressed this more clearly in the discussion and limitations section (see lines 660-663 and 690-694 of the revised manuscript).

9. The biosecurity questionnaire data collected should be presented descriptively in a supplementary table, and authors should acknowle that the inability to include biosecurity data as a covariate is a major limitation of the study design. Please update limitations section.

Further information on other livestock reared on each farm, assessment of some hygiene measures and comments have been added as new **Supplementary Material S1** and a small dedicated section in the methods (lines 151-158 revised manuscript).
The limitations section was updated (see lines 694-699 revised manuscript). We agree that inclusion of biosecurity data, especially standardized and weighted biosecurity scores, such as Biocheck.Ugent (https://biocheckgent.com/en), would be a major improvement that need to be applied to future studies. While there are no own covariates for different biosecurity and management factors on each farm, the random intercept for each farm (see comment 6 for modelling regarding random intercept for farm 8) can be assumed to capture such farm specific aspects to some degree in our study.

10. Line 403: "Isolates not assigned to ST829-CC" typo for "ST828-CC."?

Thank you for bringing it to our attention, we have corrected this.

11. Lines 540-541 state that "all CIP-R Campylobacter isolates carried the gyrA T86I mutation. However, later in the manuscript it is reported that one CIP-R isolate with a MIC of 1 mg/L where gyrA T86I was not detected. Please harmonize.

Thank you for pointing this out, we have rephrased the sentence accordingly (line 558 of revised manuscript).

12. The authors state that MLST and AMR screening were performed using the BakCharak pipeline (gitlab.com/bfr_bioinformatics/bakcharak), but the description provided is insufficient for reproducibility. Please clarify: 1. the version of BakCharak. 2. since the MLST module within BakCharak relies on SISTR it is unclear which tool and allele database were used for Campylobacter MLST. 3. Confirm AMRFinder was used using the organism specific mode (--organism Campylobacter); also confirm AMRFinder database version used.

Please note that the Bakcharak version 3.0.3 was mentioned in line 237 of the original manuscript (line 245 revised manuscript). The mlst module 2.23.0 within the Bakcharak pipeline uses the

PubMLST database (last update 2025-02-27) as a reference. All analysis on AMR and MLST have been conducted with the species-specific internal *Campylobacter* pipeline of Bakcharak, ensuring that AMRFinder version 3.12.8 (last update 2024-01-31) was used in the organism specific mode.

An analysis through the ResFinder database 4.4.1 has been additionally conducted. All information were added to the methods section (see lines 252-258 revised manuscript).

Reviewer #3 (Comments for the Author):

Manuscript entitled "Longitudinal ......farms" is well written. The experiments are well designed and it is an interesting and a timely article.

We thank reviewer #2 for their evaluation of the manuscript and their kind feedback.

Reviewer #4 (Comments for the Author):
The manuscript (control no. Spectrum 01012-26) entitled "Longitudinal study of prevalence and antimicrobial resistance of thermotolerant Campylobacter spp. in German organic meat chicken farms". This study aimed to examine the prevalence and antimicrobial resistance of thermotolerant Campylobacter spp. in fourteen German organic meat chicken farms rearing slow-growing broiler, male layer hybrids or dual-purpose cockerels in a longitudinal study.
The results revealed that at the end of the fattening period, 98 % of all flocks were positive with C. jejuni being the predominant species. Antimicrobial resistance of 405 C. jejuni and 79 C. coli revealed overall high levels of ciprofloxacin and tetracycline resistance but absence of macrolide resistance. Results for flocks within farms showed clustering effects (ICCCIP = 0.447; ICCTET = 0.412). Whole genome sequencing of representative isolates confirmed great overall genetic variety, with farm- or fattening type-dependent dissemination of certain multi-locus sequence types. The majority of C. jejuni belonged to ST21 clonal complex. The majority of C. coli belonged to ST828 clonal complex.

This manuscript is well written, the experiment is well designed and the results is correctly illustrated in different table and informative figures.

We thank reviewer #1 for their positive evaluation of the manuscript and the provided comments which helped to further improve our manuscript. We have addressed all recommendations of this reviewer in the revised version as listed below.

General comment:
1. Although this manuscript described an essential research point for a significant foodborne pathogen in organic meat chicken farms, this study did not discuss all epidemiological aspects of the farms and if there is any contact with other animals might be as a source of transmission.

Please see the answer for reviewer #1 comment 9 above for a detailed answer; further information of other livestock on farm has been added to the supplements and a limitations section was added to the discussion, where these points were expanded upon.
We have discussed contact with animals as a potential source of transmission in lines 472-474 of the original manuscript, but did not sample other livestock for *Campylobacter* isolation and

characterisation, so we have no valid data to examine and discuss their relevance for our study in detail.

2. This study performed in organic poultry farms and the authors (lines 153-156) showed the antimicrobial treatment of flocks, could you briefly expand this point and show the reason of medication to give the reader have an overview about the previously published data on the same flocks.

The indications of all treatments were included (lines 154-158 of the revised manuscript).

3. In the title, the authors give us a quick feeling that the samples were collected from slaughterhouses "German organic meat chicken farms", it will be better if you replace it either with broilers or poultry farms.

We see why the word “meat chicken farm” could be initially confusing and would like to explain our reasoning. “Meat chicken” was used instead of “broiler” to address the inclusion of chicken genotypes reared for fattening other than fattening hybrids. The word “broiler” is strongly associated or synonymously used with “fattening hybrid” in Germany (and to our understanding other parts of the world), which is why it has been also used for the group of slow-growing fattening hybrids in tables and figures of the manuscript as well as different parts of the discussion. “Poultry” seemed too unspecific to us, as this could include other species like turkeys, geese and ducks. As the sampling scheme at farm level is clearly explained in the abstract, we would prefer to keep the title as is.

4. In methodology section, the author needs to make the study design more concise and don't build on the previous part of the work which previously published elsewhere. It means, the reader needs to read the previous publication to understand the current one.

The sampling scheme and study population was the same as in our previous study on *E. coli*. Accordingly, detailed (supplementary) tables regarding the flocks and hatcheries have already been published under open access in *Poultry Science*. All important details were summarized for this article in accordance with common scientific writing practice, as we cannot reproduce previously published tables in compliance with Good Scientific Practice. This would be redundant and may raise copyright concerns. As we provided supplementary materials (**S2**) with complete metadata for each isolate, all data are also available within this publication.

5. In line 162: please add the method to adjust the microaerophilic condition.

The methods were added in lines 170-173 of the revised manuscript.

6. In the result section (Lines 276-280), considered as part of methodology section.

We have moved this paragraph to the methods section under “study population and sampling time points” (lines 143-147 revised manuscript).

7. In spite of the manuscript is well written and informative, there are some grammatic and typing errors in the manuscript. The authors should revise the whole manuscript for these minimal typing mistakes (e.g. lines 17; 20; 29....).

Thank you for bringing it to our attention, we have thoroughly scanned the manuscript and hope to have eliminated all last mistakes.

8. In the result section "Resistance determinants and phenotypical resistance in Campylobacter", line 434: the authors used AMRFinder database, please clarify why only this tool used and not others as ResFinder or NCBI, etc......

Please also refer to reviewer #1 comment 5 and 12 for added analyses and changes to the manuscript regarding resistance determinants.

Isolates have been now been additionally analyzed with the ResFinder 4.4.1 database and discrepancies noted in **Supplementary Material S2**. Other than the flagging of 50S_L22_A103V in AMRFinder as a, 36 isolates were identified that carried *tet*(O32O), which was previously identified as tet(O) with reduced identity scores (93%) by AMRFinder.

9. The discussion section is very long and need to be summarized.

We have streamlined some of the original wording, but were asked to expand on several central points by reviewer #1 and therefore could not shorten the discussion as a whole.

10. In Table 1: in the raw of slow growing flock, the total positive was 18 out 20 but in the column of C. jejuni and C. coli were mire than 18. Please clarify.

That is correct and we have rechecked all data to verify this.
As described in lines 291-292 of the original manuscript (314-315 revised manuscript), both *C. jejuni* and *C. coli* could be isolated from 17.8 % (24/135) of samples. For example: In S3 slow-growing broilers, 16/20 samples were positive for *C. jejuni* only and 2/20 for *C. coli* only (18/20 samples overall positive for Campylobacter spp.). Of the 16 samples positive for *C. jejuni*, two showed additional growth for *C. coli* (4/20 samples positive for *C. coli* in total as mentioned in the table). This principle affects some other rows of Table 1 as well, but is sufficiently explained by the text and by giving the total of *Campylobacter* positive samples per time point and group. Furthermore, the whole dataset on all isolates and metadata is available through **Supplementary Material S2 Table 1.**

Comparison of GLMM results with and without isolates of farm 8

GLMM orig.: previously submitted final model and results of statistical analysis
GLMM comp.: results of the same model with exclusion of isolates from farm 8 for comparison

Substantial differences in significance (significant vs. non-significant) upon exclusion of farm 8 are highlighted in yellow

CIP-R *C. jejuni:*

		GLMM orig	GLMM comp
**Case Processing Summary**	included	405	376
	excluded	0	0
	total	405	376
**Model Summary**	target	CIP_R	CIP_R
	Probability distribution	binominal	binominal
	link function	logit	logit
**Model Fitness**	Akaike corrected (AIC)	2225.505	2077.897
	Bayesian (BIC)	2233.417	2085.652
	-2 Log-Likelihood	2221.474	2073.863
	Pseudo-R2 measure (marginal)	0.189	0.183
	Pseudo-R2 measure (conditional)	0.733	0.749
**Intraclass Correlation Coefficients**	Overall ICC (adjusted)	0.671	0.692
	Overall ICC (conditional)	0.544	0.565
	Farm ICC (adjusted)	0.120	0.131
	Farm ICC (conditional)	0.098	0.107
	Farm*Flock ICC (adjusted)	0.551	0.561
	Farm*Flock ICC (conditional)	0.447	0.458
	Classification (overall)	92.6%	92.0%
	Sensitivity	96.4%	95.6%
	Specificity	86.4%	86.6%

Fixed Effects GLMM orig.

**Source**	**F**	**df1**	**df2**	**Sig.**
**Corrected Model**	3.308	12	392	**<0.001** **
**Season**	5.170	3	392	**0.002** *
**Fattening type**	1.973	2	392	0.140
**Sampling time point**	0.222	2	392	0.801
**Fattening type*sampling time point**	4.619	4	392	**0.001** *
**AB treatment**	0.678	1	392	0.411

Fixed Effects GLMM comp.

**Source**	**F**	**df1**	**df2**	**Sig.**
**Corrected Model**	2.991	12	363	**0.001** *
**Season**	4.411	3	363	**0.005** *
**Fattening type**	1.297	2	363	0.275
**Sampling time point**	0.083	2	363	0.921
**Fattening type*sampling time point**	4.769	4	363	**0.001** *
**AB treatment**	0.572	1	363	0.450

TET-R *C. jejuni*:

		GLMM orig	GLMM comp
**Case Processing Summary**	included	405	376
	excluded	0	0
	total	405	376
**Model Summary**	target	TET_R	TET_R
	Probability distribution	binominal	binominal
	link function	logit	logit
**Model Fitness**	Akaike corrected (AIC)	2173. 947	2017.029
	Bayesian (BIC)	2181. 858	2024.784
	-2 Log-Likelihood	2169.916	2012.996
	Pseudo-R2 measure (marginal)	0.254	0.255
	Pseudo-R2 measure (conditional)	0.753	0.755
**Intraclass Correlation Coefficients**	Overall ICC (adjusted)	0.669	0.671
	Overall ICC (conditional)	0.499	0.500
	Farm ICC (adjusted)	0.116	0.078
	Farm ICC (conditional)	0.087	0.058
	Farm*Flock ICC (adjusted)	0.553	0.592
	Farm*Flock ICC (conditional)	0.412	0.441
	Classification (overall)	90.9%	90.4%
	Sensitivity	96.4%	94.2%
	Specificity	81.7%	84.9%

Fixed Effects GLMM orig.

**Source**	**F**	**df1**	**df2**	**Sig.**
**Corrected Model**	3.960	12	392	**<0.001 ****
**Season**	6.828	3	392	**<0.001 ****
**Fattening type**	3.477	2	392	**0.032** *
**Sampling time point**	0.915	2	392	0.401
**Fattening type*sampling time point**	8.898	4	392	**<0.001 ****
**AB treatment**	0.014	1	392	0.904

Fixed Effects GLMM comp.

**Source**	**F**	**df1**	**df2**	**Sig.**
**Corrected Model**	3.868	12	363	**<0.001 ****
**Season**	6.943	3	363	**<0.001 ****
**Fattening type**	2.094	2	363	0.125
**Sampling time point**	1.257	2	363	0.286
**Fattening type*sampling time point**	9.161	4	363	**<0.001 ****
**AB treatment**	0.007	1	363	0.932

Pairwise comparisons without farm 8

Pairwise contrasts of fixed effects season, fattening type, sampling time point and antimicrobial treatment from mixed effects logistic regression

		CIP resistance C. jejuni					TET resistance C. jejuni				
		CE	OR	95% CI		p[a)]	CE	OR	95% CI		p[a)]
				Lower	Upper				Lower	Upper	
Season	Spring vs. Summer	0,1	1,1	0,2	5,5	1,000	-1,4	0,3	0,0	1,8	0,285
	Spring vs. Autumn	-2,1	0,1	0,0	1,5	0,145	-1,1	0,3	0,0	2,9	0,513
	Spring vs. Winter	0,6	1,8	0,1	25,1	1,000	**2,9**	**18,0**	**1,4**	**224,2**	**0,017***
	Summer vs. Winter	0,5	1,7	0,2	16,8	1,000	**4,3**	**71,5**	**5,2**	**973,0**	**<0,001****
	Autumn vs. Summer	**2,1**	**8,6**	**1,2**	**59,7**	**0,021***	-0,3	0,7	0,2	2,8	0,657
	Autumn vs. Winter	**2,7**	**14,5**	**1,2**	**175,5**	**0,029***	4,0	53,1	4,6	616,9	**<0,001****
Fattening type	Broiler vs. Male layer	0,5	1,7	0,1	27,5	0,710	1,5	4,3	0,2	76,6	0,501
	Dual-purpose vs. Broiler	2,0	7,4	0,2	233,5	0,388	1,5	4,3	0,2	83,6	0,501
	Dual-purpose vs. Male layer	2,5	12,5	0,2	641,3	0,372	2,9	18,8	0,6	592,7	0,125
Sampling time point	S3 vs. S4	0,1	1,1	0,3	4,5	1,000	0,8	2,3	0,6	8,6	0,394
	S3 vs. S5	0,3	1,3	0,3	6,6	1,000	0,7	2,1	0,6	7,4	0,394
	S4 vs. S5	0,2	1,2	0,4	3,9	1,000	-0,1	0,9	0,4	2,2	0,852
AB treatment	AB treatment vs. No AB	1,0	2,7	0,2	37,7	0,450	0,1	1,2	0,0	36,1	0,932

*a) sequential Bonferroni adjusted p; * significant < 0.05, ** highly significant < 0.001*

Pairwise contrasts of fattening types at sampling time points S3-S5 from mixed effects logistic regression

Time point	Fattening types	CIP Resistance C. jejuni					TET Resistance C. jejuni				
		CE	OR	95% CI Lower	95% CI Upper	p[a)]	CE	OR	95% CI Lower	95% CI Upper	p[a)]
S3	Broiler vs. Male layer	1,9	6,9	0,3	178,0	0,243	**3,7**	**40,3**	**2,0**	**820,8**	**0,016***
	Dual-purpose vs. Broiler	3,9	51,8	0,6	4311,1	0,091	**4,5**	**91,3**	**2,3**	**3617,0**	**0,012***
	Dual-purpose vs. Male layer	**5,9**	**357,7**	**1,8**	**69923,8**	**0,023***	**8,2**	**3678,6**	**43,3**	**312582,6**	**<0,001****
S4	Broiler vs. Male layer	1,4	4,2	0,1	173,9	1,000	2,3	10,2	0,4	263,8	0,218
	Dual-purpose vs. Broiler	-1,2	0,3	0,0	17,5	1,000	0,6	1,8	0,1	32,3	0,697
	Dual-purpose vs. Male layer	0,3	1,3	0,0	55,3	1,000	2,9	18,1	0,4	782,3	0,195
S5	Broiler vs. Male layer	-1,8	0,2	0,0	5,5	0,503	-1,7	0,2	0,0	5,6	0,645
	Dual-purpose vs. Broiler	3,2	25,0	0,4	1610,0	0,191	1,8	5,8	0,2	176,9	0,645
	Dual-purpose vs. Male layer	1,4	4,2	0,1	159,3	0,503	0,0	1,0	0,1	19,9	0,984

*a) sequential Bonferroni adjusted p; * significant < 0.05, ** highly significant < 0.001*

Pairwise contrasts of sampling time points within fattening types from mixed effects logistic regression

		CIP Resistance C. jejuni					TET Resistance C. jejuni				
Fattening types	Sampling time point	CE	OR	95% CI		p[a)]	CE	OR	95% CI		p[a)]
				Lower	Upper				Lower	Upper	
Broiler	S3 vs. S4	-1,5	0,2	0,0	1,2	0,103	-0,2	0,8	0,2	3,1	0,744
	S3 vs. S5	1,2	3,5	0,8	15,8	0,106	1,6	5,1	1,0	26,6	0,057
	S4 vs. S5	**2,7**	**14,9**	**1,8**	**126,7**	**0,008***	**1,8**	**6,3**	**1,1**	**35,8**	**0,032***
Male layer	S3 vs. S4	-2,0	0,1	0,0	1,5	0,119	**-1,7**	**0,2**	**0,0**	**1,0**	**0,045***
	S5 vs. S3	2,5	11,8	1,0	141,1	0,052	**3,9**	**51,8**	**5,1**	**523,2**	**<0,001****
	S5 vs. S4	0,5	1,7	0,4	7,1	0,495	**2,3**	**9,7**	**1,8**	**53,0**	**0,005***
Dual-purpose	S3 vs. S4	3,7	39,0	0,6	2501,9	0,104	**4,4**	**80,4**	**4,3**	**1495,0**	**0,002***
	S3 vs. S5	2,0	7,2	0,2	238,7	0,376	**4,6**	**95,9**	**5,1**	**1816,6**	**0,001***
	S4 vs. S5	-1,7	0,2	0,0	3,3	0,376	0,2	1,2	0,2	6,9	0,845

*a) sequential Bonferroni adjusted p; * significant < 0.05, ** highly significant < 0.001*

Re: Spectrum01012-26R1 (Longitudinal study of prevalence and antimicrobial resistance of thermotolerant Campylobacter spp. in German organic meat chicken farms)

Dear Dr. Anna Maria Korves-Wilm:

Your manuscript has been accepted, and I am forwarding it to the ASM production staff for publication. Your paper will first be checked to make sure all elements meet the technical requirements. ASM staff will contact you if anything needs to be revised before copyediting and production can begin. Otherwise, you will be notified when your proofs are ready to be viewed.

Data Availability: ASM policy requires that data be available to the public upon online posting of the article, so please verify all links to sequence records, if present, and make sure that each number retrieves the full record of the data. If a new accession number is not linked or a link is broken, provide production staff with the correct URL for the record. If the accession numbers for new data are not publicly accessible before the expected online posting of the article, publication may be delayed; please contact ASM production staff immediately with the expected release date.

Publication Fees: For information on publication fees and which article types have charges, please visit our website. We have partnered with Copyright Clearance Center (CCC) to collect author charges. If fees apply to your paper, you will receive a message from no-reply@copyright.com with further instructions. For questions related to paying charges through RightsLink, please contact CCC at ASM_Support@copyright.com or toll free at +1-877-622-5543. CCC makes every attempt to respond to all emails within 24 hours.

ASM Membership: Corresponding authors may join or renew ASM membership to obtain discounts on publication fees. Need to upgrade your membership level? Please contact Customer Service at Service@asmusa.org.

Embargo Policy: A press release may be issued as soon as the manuscript is posted on the Spectrum Latest Articles webpage. The corresponding author will receive an email with the subject line "ASM Journals Author Services Notification" when the article is available online.

Cover Image Submissions: If you would like to submit a potential Cover Image, please email a file and a short legend to spectrum@asmusa.org. Please note that we can only consider images that (i) the authors created or own and (ii) have not been previously published. By submitting, you agree that the image can be used under the same terms as the published article. Image File requirements: TIF/EPS, 7.5 inches wide by 8.25 inches tall (at least 2,250 pixels wide by 2,475 pixels tall), minimum 300 dpi resolution (600 dpi preferred), RGB, and no figure elements, e.g., arrows or panel labels. The legend should be a short description of the image, 1-2 sentences recommended. Please use the ASM Journals Cover Image Sizing Tool (https://journals.asm.org/pb-assets/html/asm-cover-sizing-tool.html) to ensure that your proposed cover image meets our size requirements.

The ASM Journals program strives for constant improvement in our submission and publication process. Please tell us how we can improve your experience by taking this quick Author Survey.

Thank you for submitting your paper to Spectrum.

Sincerely,
Orhan Sahin
Editor
Microbiology Spectrum
