# Supplementary material for: Longitudinal study of prevalence and antimicrobial resistance of thermotolerant Campylobacter spp. in German organic meat chicken farms
Source: Microbiol Spectr. 2026 Sep 4;14(10):e01012-26. doi: 10.1128/spectrum.01012-26 (PMC13689127; doi:10.1128/spectrum.01012-26)
Supplement: Supplementary file 2 [file spectrum.01012-26-s0001.pdf]

File S1: Longitudinal study of prevalence and antimicrobial resistance of thermotolerant *Campylobacter* spp.in German organic meat chicken farms

Anna Maria Korves-Wilm[1], Kerstin Stingl[1], Margret Krieger[2], Mirjam Grobbel[1], Bernd-Alois Tenhagen[1]

Authors Affiliations:

[1]Department Biological Safety, German Federal Institute for Risk Assessment, Berlin, Germany

[2]University of Kassel, Witzenhausen, Germany

Farm information and biosecurity questionnaire

	Farm 1	Farm 2	Farm 3	Farm 4	Farm 5	Farm 6	Farm 7	Farm 8	Farm 9	Farm 10	Farm 11	Farm 12	Farm 13	Farm 14
**Fattening type**	broiler	broiler	broiler	dual-purpose	male layer	dual-purpose	broiler	dual-purpose	dual-purpose	broiler	male layer	male layer	male layer	male layer
**Farm area (ha)**	150	90	9	91	104	2,5	13,2	*n.a.*	120	80	*n.a.*	50	111	100
**Other livestock on farm**	laying hens, geese	cattle, pig	none	cattle, pigs, laying hens, ducks, geese, goats	laying hens	turkeys, laying hens, ducks	none	dairy cows, laying hens	laying hens	cattle, laying hens *	laying hens	cattle, laying hens	laying hens	laying hens
**Shared pastures [a)]**	no	no	no	yes, with goats	no	no	yes, with goats	no	no	no	no	yes, cattle and chicken in rotation	no	no
**Water source**	city water	well	city water	both	well	city water	city water	city water	well	city water	city water	well	city water	city water
**Watersystem cleaning frequency (times per month) [b)]**	0	0	1	0	0	0	0	0	4	4	0	2	0	1
**Rodent control [c)]**	yes	yes	yes	yes	yes	yes	yes	no	yes	yes	yes	yes	yes	yes
**Same caretaker for different poultry on farm**	yes	yes	no	*n.a.*	yes	yes	no	no	yes	yes	yes	yes	yes	yes
**Caretaker has poultry at home**	yes	*n.a.*	no	*n.a.*	no	yes	yes	yes	no	no	no	no	yes	no
**Separate clothing**	no	no	no	per stable	no	no	per poultry area	no	no	no	per stable	per stable	no	no
**Separate shoes**	no	per stable	per stable	per stable	no	per stable	per stable	no	per stable	per stable	per stable	per stable	per stable	per poultry area
**Hand wash**	yes	yes	no	no	no	no	yes	no	yes	no	no	yes	yes	yes
**Hand desinfection**	no	yes	yes	yes	no	no	no	*n.a.*	no	no	no	yes	no	yes

	Farm 1	Farm 2	Farm 3	Farm 4	Farm 5	Farm 6	Farm 7	Farm 8	Farm 9	Farm 10	Farm 11	Farm 12	Farm 13	Farm 14
**Desinfection mat and/or tub**	no	no	no	no	yes	yes	no	no	no	yes	yes	yes	yes	yes
**Hygiene sluice stable**	yes	yes	no	no	no	yes	yes	no	no	yes	no	yes	no	no
**Hygiene sluice farm**	no	no	no	no	yes	no	no	no	yes	no	no	no	no	yes
**Hygiene concept for visitors**	*n.a.*	no	no	*n.a.*	yes	yes	yes	yes	yes	no	*n.a.*	yes	yes	yes
**Duration of service period (days)**	*n.a.*	17	14	14	30	14	7	27	14	10	14	23	17	10
**Litter removal frequency [d)]**	during fattening if necessary	after each flock	after each flock	three times during fattening	after each flock	after each flock	after each flock	after each flock	after each flock	after each flock	after each flock	after each flock	after each flock	during fattening if necessary
**R&D service period: dry or wet cleaning?**	dry	wet	dry	*n.a.*	wet	wet	wet	wet	wet	wet	wet	wet	wet	wet
**R&D service period: Removal of litter**	yes.	yes	yes	yes	yes	yes	yes	yes	yes	yes	yes	yes	yes	yes
**R&D service period: cleaning with waterhose**	no	no	no	no	yes	yes	no	yes	no	no	yes	yes	no	no
**R&D service period: high pressure cleaner**	every two to three flocks	yes	no	yes	yes	yes	yes	yes	yes	yes	yes	yes	yes	yes
**R&D service period: desinfection**	no	yes	yes	yes	yes	yes	yes	no	yes	yes	yes	yes	yes	no
**R&D service period: desinfection of outdoorrun [e)]**	no	no	yes	no	no	no	no	no	no	yes	no	no	no	yes

* broilers are kept on a separate location 600 m apart from cattle and laying hens

a) simultaneous pasturing in the same outdoor run; rotation of outdoor runs between different species was specified in conversation with the caretaker

b) times per month; 0 represents a less than once a month frequency

c) method and frequency of rodent control was not specified and could not be verified

d) answers “weekly”, “monthly”, “after each flock” or “other” with the latter specified in free text answers or conversation with the caretaker

e) with quicklime
