# Supplementary material for: Longitudinal study of prevalence and antimicrobial resistance of thermotolerant Campylobacter spp. in German organic meat chicken farms
Source: Microbiol Spectr. 2026 Sep 4;14(10):e01012-26. doi: 10.1128/spectrum.01012-26 (PMC13689127; doi:10.1128/spectrum.01012-26)
Supplement: Supplementary file 3 [file spectrum.01012-26-s0002.pdf]

File S2: Longitudinal study of prevalence and antimicrobial resistance of thermotolerant *Campylobacter* spp.in German organic meat chicken farms

Anna Maria Korves-Wilm*[1], Kerstin Stingl[1], Margret Krieger[2], Mirjam Grobbel[1], Bernd-Alois Tenhagen[1]

Authors Affiliations:

[1] Department Biological Safety, German Federal Institute for Risk Assessment, Berlin, Germany

[2] University of Kassel, Witzenhausen, Germany

Generalized linear mixed models – Model summary “Ciprofloxacin resistant *C. jejuni*”

Null model: intercept only model, includes random intercepts for farm and farm nested in flock (farm*flock)

GLMM 1: model containing fixed effects and random intercepts

GLMM 2: final model containing fixed effects with interaction fattening type*sampling time point and random intercepts

		Nullmodell	GLMM 1	GLMM 2
**Case Processing Summary**	**included**	405	405	405
	**excluded**	0	0	0
	**total**	405	405	405
**Model Summary**	**target**	CIP_R	CIP_R	CIP_R
	**Probability distribution**	binominal	binominal	binominal
	**link function**	logit	logit	logit
**Model Fitness**	**Akaike corrected (AICc)**	2014.027	2199.396	2225.505
	**Bayesian (BIC)**	2022.000	2207.328	2233.417
	**-2 Log-Likelihood**	2009.997	2195.365	2221.474
	**Pseudo-R^2 measure (marginal)**	0	0.158	0.189
	**Pseudo-R^2 measure (conditional)**	0.565	0.701	0.733
**Intraclass Correlation Coefficients**	**Overall ICC (adjusted)**	0.565	0.645	0.671
	**Overall ICC (conditional)**	0.565	0.543	0.544
	**Farm ICC (adjusted)**	0.157	0.157	0.12
	**Farm ICC (conditional)**	0.157	0.132	0.098
	**Farm*flock ICC (adjusted)**	0.408	0.488	0.551
	**Farm*flock ICC (conditional)**	0.408	0.410	0.447
	**Classification (overall)**	86.7%	91.6%	92.6%
	**Sensitivity**	95.2%	94.4%	96.4%
	**Specificity**	72.7%	87%	86.4%

“Ciprofloxacin resistant *C. jejuni*” Final model – Fixed effects

**Source**	**F**	**df1**	**df2**	**Sig.**
Corrected Model	3.308	12	392	0.000
Season	5.170	3	392	0.002
Fattening type	1.973	2	392	0.140
Sampling time point	0.222	2	392	0.801
Fattening type*sampling time point	4.619	4	392	0.001
AB treatment	0.678	1	392	0.411

"Ciprofloxacin resistant *C. jejuni*" Final model – Fixed Coefficients

Model Term	Coefficient	Std. Error	t	Sig.	95% Confidence Interval		Exp(Coefficient)	95% Confidence Interval for Exp(Coefficient)	
					Lower	Upper		Lower	Upper
**Intercept**	3.059	1.6558	1.847	0.065	-0.196	6.314	21.307	0.822	552.443
**Spring**	0.487	0.9948	0.489	0.625	-1.469	2.442	1.627	0.230	11.501
**Summer**	0.158	0.9532	0.166	0.868	-1.716	2.032	1.171	0.180	7.629
**Autumn**	2.489	0.9069	2.744	0.006	0.706	4.272	12.044	2.025	71.639
**Winter**	0	-	-	-	-	-	-	-	-
**Slow-growing broiler**	-3.880	1.8466	-2.101	0.036	-7.511	-0.249	0.021	0.001	0.779
**Male layer hybrids**	-5.664	2.0690	-2.738	0.006	-9.732	-1.597	0.003	5.936×10^{-5}	0.203
**Dual-purpose cockerels**	0	-	-	-	-	-	-	-	-
**Sampling time point S4**	-2.970	1.5981	-1.859	0.064	-6.112	0.172	0.051	0.002	1.187
**Sampling time point S5**	-1.791	1.5111	-1.185	0.237	-4.762	1.180	0.167	0.009	3.253
**Sampling time point S3**	0	-	-	-	-	-	-	-	-
**Broiler * S4**	4.436	1.7468	2.539	0.011	1.002	7.870	84.439	2.723	2618.534
**Broiler * S5**	0.556	1.6708	0.333	0.740	-2.729	3.841	1.743	0.065	46.550
**Broiler * S3**	0	-	-	-	-	-	-	-	-
**Male layer * S4**	4.790	1.9309	2.481	0.014	0.994	8.586	120.310	2.702	5357.927
**Male Layer * S5**	4.155	1.8553	2.239	0.026	0.507	7.802	63.720	1.660	2445.571
**Male Layer * S3**	0	-	-	-	-	-	-	-	-
**Dual Purpose * S4**	0	-	-	-	-	-	-	-	-
**Dual Purpose * S5**	0	-	-	-	-	-	-	-	-
**Dual purpose * S3**	0	-	-	-	-	-	-	-	-
**With AB treatment**	1.092	1.3255	0.824	0.411	-1.514	3.698	2.979	0.220	40.348
**Without AB treatment**	0	-	-	-	-	-	-	-	-

Generalized linear mixed models – Model summary "Tetracycline resistant *C. jejuni*"

Null model: intercept only model, includes random intercepts for farm and farm nested in flock (farm*flock)

GLMM 1: model containing fixed effects and random intercepts

GLMM 2: final model containing fixed effects with interaction fattening type*sampling time point and random intercepts

		Nullmodell	GLMM 1	GLMM 2
**Case Processing Summary**	**included**	405	405	405
	**excluded**	0	0	0
	**total**	405	405	405
**Model Summary**	**target**	TET_R	TET_R	TET_R
	**Probability distribution**	binominal	binominal	binominal
	**link function**	logit	logit	logit
**Model Fitness**	**Akaike corrected (AICc)**	1950.261	2005.779	2173. 947
	**Bayesian (BIC)**	1958.234	2013.771	2181. 858
	**-2 Log-Likelihood**	1946.231	2001.748	2169.916
	**Pseudo-R^2 measure (marginal)**	0.000	0.139	0.254
	**Pseudo-R^2 measure (conditional)**	0.494	0.596	0.753
**Intraclass Correlation Coefficients**	**Overall ICC (adjusted)**	0.494	0.531	0.669
	**Overall ICC (conditional)**	0.494	0.457	0.499
	**Farm ICC (adjusted)**	0.144	0.108	0.116
	**Farm ICC (conditional)**	0.144	0.093	0.087
	**Farm*flock ICC (adjusted)**	0.350	0.423	0.553
	**Farm*flock ICC (conditional)**	0.350	0.364	0.412
	**Classification (overall)**	84.0%	86.4%	90.9%
	**Sensitivity**	94.4%	93.7%	96.4%
	**Specificity**	66.7%	74.5%	81.7%

"Tetracycline resistant *C. jejuni*" Final model – Fixed effects

**Source**	**F**	**df1**	**df2**	**Sig.**
Corrected Model	3.960	12	392	0.000
Season	6.828	3	392	0.000
Fattening type	3.477	2	392	0.032
Sampling time point	0.915	2	392	0.401
Fattening type*sampling time point	8.898	4	392	0.000
AB treatment	0.014	1	392	0.904

“Tetracycline resistant *C. jejuni*” Final model – Fixed Coefficients

Model Term	Coefficient	Std. Error	t	Sig.	95% Confidence Interval		Exp(Coefficient)	95% Confidence Interval for Exp(Coefficient)	
					Lower	Upper		Lower	Upper
**Intercept**	2.393	1.3371	1.790	0.074	-0.236	5.022	10.947	0.790	151.676
**Spring**	2.721	0.9497	2.865	0.004	0.854	4.588	15.198	2.349	98.332
**Summer**	3.971	0.9450	4.202	0.000	2.113	5.829	53.044	8.275	340.002
**Autumn**	3.679	0.8660	4.249	0.000	1.977	5.382	39.623	7.220	217.439
**Winter**	0	-	-	-	-	-	-	-	-
**Slow-growing broiler**	-4.515	1.6350	-2.761	0.006	-7.729	-1.300	0.011	0.000	0.273
**Male layer hybrids**	-8.210	1.8477	-4.444	0.000	-11.843	-4.578	0.000	7.190×10^{-6}	0.010
**Dual-purpose cockerels**	0	-	-	-	-	-	-	-	-
**Sampling time point S4**	-3.704	1.2286	-3.015	0.003	-6.119	-1.288	0.025	0.002	0.276
**Sampling time point S5**	-4.335	1.1602	-3.737	0.000	-6.616	-2.054	0.013	0.001	0.128
**Sampling time point S3**	0	-	-	-	-	-	-	-	-
**Broiler * S4**	3.940	1.3823	2.850	0.005	1.222	6.658	51.416	3.395	778.655
**Broiler * S5**	2.752	1.3510	2.037	0.042	0.096	5.408	15.669	1.100	223.160
**Broiler * S3**	0	-	-	-	-	-	-	-	-
**Male layer * S4**	5.313	1.5312	3.470	0.001	2.302	8.323	202.894	9.996	4118.063
**Male Layer * S5**	8.180	1.5940	5.132	0.000	5.046	11.314	3569.062	155.417	81961.593
**Male Layer * S3**	0	-	-	-	-	-	-	-	-
**Dual Purpose * S4**	0	-	-	-	-	-	-	-	-
**Dual Purpose * S5**	0	-	-	-	-	-	-	-	-
**Dual purpose * S3**	0	-	-	-	-	-	-	-	-
**With AB treatment**	0.210	1.7474	0.120	0.904	-3.225	3.646	1.234	0.040	38.314
**Without AB treatment**	0	-	-	-	-	-	-	-	-
