# Supplementary material for: Longitudinal study of prevalence and antimicrobial resistance of thermotolerant Campylobacter spp. in German organic meat chicken farms
Source: Microbiol Spectr. 2026 Sep 4;14(10):e01012-26. doi: 10.1128/spectrum.01012-26 (PMC13689127; doi:10.1128/spectrum.01012-26)
Supplement: Supplementary file 4 [file spectrum.01012-26-s0003.pdf]

File S3: Longitudinal study of prevalence and antimicrobial resistance of thermotolerant *Campylobacter* spp. in German organic meat chicken farms

Anna Maria Korves-Wilm*[1], Kerstin Stingl[1], Margret Krieger[2], Mirjam Grobbel[1], Bernd-Alois Tenhagen[1]

Authors Affiliations:

[1] Department Biological Safety, German Federal Institute for Risk Assessment, Berlin, Germany

[2] University of Kassel, Witzenhausen, Germany

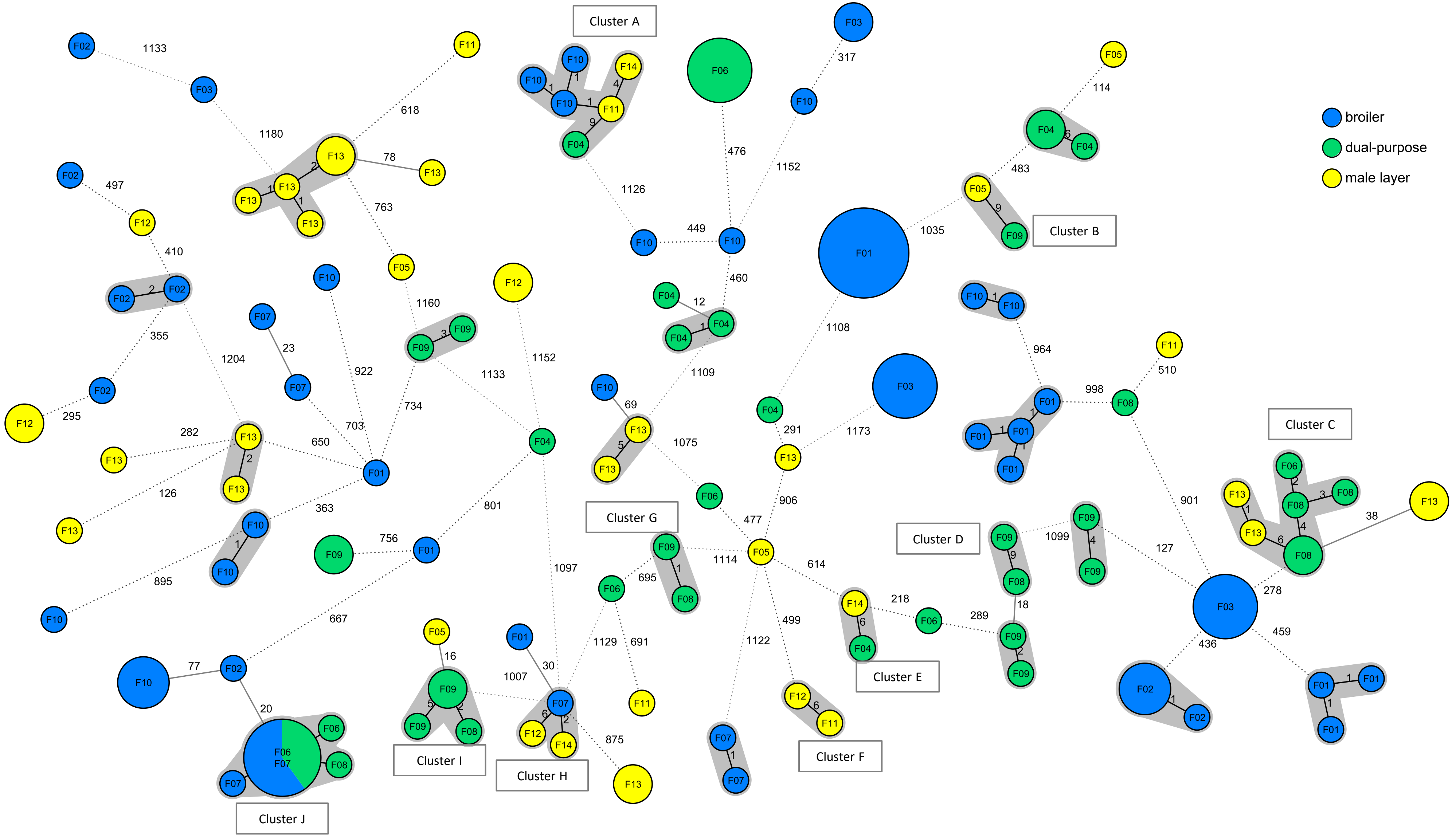

cgMLST-based minimum spanning tree for 145 *C. jejuni* constructed in Ridom SeqSphere + v10.5.2 with a scheme of 1,343 core loci, nodes annotated with farm number and fattening type (colour)

C. jejuni

Cluster	Isolates	MLST	Source	Hatchery	Season	Phenotypical resistance	Resistance determinants	Comment
A	23-EP00055-1	ST51	F11-Fl1-S5	H4	summer	susceptible	50S_L22_A103V;blaOXA-184	
	23-EP00151-1		F10-Fl3-S3	H2	autumn	susceptible	50S_L22_A103V;blaOXA-184	
	24-EP00003-4		F10-Fl4-S3	H2	winter	susceptible	50S_L22_A103V;blaOXA-184	
	24-EP00031-2		F10-Fl4-S4	H2	winter	susceptible	50S_L22_A103V;blaOXA-184	
	24-EP00103-2		F4-Fl4-S3	H3	spring	TET	50S_L22_A103V;blaOXA-184;tet(O)	
	24-EP00145-2		F14-Fl1-S3	H1	summer	susceptible	50S_L22_A103V;blaOXA-184	
B	23-EP00071-1	ST607	F5-Fl1-S4	H4	summer	susceptible	/	
	24-EP00095-3		F9-Fl2-S4	H4	spring	susceptible	/	
C	23-EP00121-3	ST50	F6-Fl2-S3	H3	autumn	CIP, TET	blaOXA-193;gyrA_T86I;tet(O)	
	23-EP00147-4		F8-Fl1-S4	H3	autumn	CIP, TET	blaOXA-193;gyrA_T86I;tet(O)	
	23-EP00171-1		F13-Fl3-S3	H4	autumn	CIP, TET	blaOXA-193;gyrA_T86I;tet(O)	
	23-EP00173-4		F13-Fl2-S5	H4	autumn	CIP, TET	blaOXA-193;gyrA_T86I;tet(O)	
	24-EP00059-1		F8-Fl2-S4	H3	spring	CIP, TET	blaOXA-193;gyrA_T86I;tet(O)	
	24-EP00153-4		F8-Fl3-S5	H4	autumn	CIP, TET	blaOXA-193;gyrA_T86I;tet(O)	
	24-EP00071-1		F8-Fl2-S5	H3	spring	CIP, TET	blaOXA-193;gyrA_T86I;tet(O)	
D	24-EP00045-4	ST10025	F9-Fl2-S3	H4	winter	CIP, TET	aph(3')-IIIa;blaOXA-193;blaOXA-61_G-57T;gyrA_T86I;sat4;tet(O)	
	24-EP00163-1		F8-Fl4-S4	H4	winter	CIP, TET	aph(3')-IIIa;blaOXA-193;blaOXA-61_G-57T;gyrA_T86I;sat4;tet(O)	
E	24-EP00103-4	ST2229	F4-Fl4-S3	H3	spring	CIP, TET	aph(3')-IIIa;blaOXA-193;blaOXA-61_G-57T;gyrA_T86I;sat4;tet(O)	
	24-EP00151-1		F14-Fl1-S4	H1	autumn	CIP, TET	aph(3')-IIIa;blaOXA-193;blaOXA-61_G-57T;gyrA_T86I;sat4;tet(O)	
F	23-EP00143-1	ST7355	F11-Fl2-S4	H4	autumn	CIP, TET	50S_L22_A103V;aph(3')-IIIa;blaOXA-193;blaOXA-61_G-57T;gyrA_T86I;sat4;tet(O)	
	24-EP00121-3		F12-Fl4_S4	H4	summer	CIP, TET	50S_L22_A103V;aph(3')-IIIa;blaOXA-193;blaOXA-61_G-57T;gyrA_T86I;sat4;tet(O)	
G	24-EP00139-2	ST2175	F9-Fl4_S4	H4	summer	TET	blaOXA-461;tet(O)	
	24-EP00141-1		F8-Fl3-S4	H4	summer	TET	blaOXA-461;tet(O)	
H	23-EP00191-4	ST5011	F7-Fl2-S4	H2	autumn	CIP, TET	blaOXA-184;gyrA_T86I;tet(O)	
	24-EP00131-1		F12-Fl4-S5	H4	summer	CIP, TET	blaOXA-184;gyrA_T86I;tet(O)	
	24-EP00157-1		F14-Fl1-S5	H1	autumn	CIP, TET	blaOXA-184;gyrA_T86I;tet(O)	
I	24-EP00055-1	ST2153	F9-Fl1-S5	H4	spring	CIP, TET	blaOXA-630;gyrA_T86I;tet(O)	
	24-EP00115-4		F9-Fl2-S5	H4	summer	CIP, TET	blaOXA-630;gyrA_T86I;tet(O)	
	24-EP00135-4		F9-Fl3-S5	H4	summer	CIP, TET	blaOXA-630;gyrA_T86I;tet(O)	
	25-EP00001-4		F8-Fl4-S5	H4	winter	CIP, TET	blaOXA-630;gyrA_T86I;tet(O)	
J	23-EP00059-1	ST9897	F7-Fl1-S3	H2	summer	CIP, TET	blaOXA-193;blaOXA-61_G-57T;gyrA_T86I;tet(O)	
	23-EP00137-3		F7-Fl2-S3	H2	autumn	CIP, TET	blaOXA-193;blaOXA-61_G-57T;gyrA_T86I;tet(O)	
	23-EP00207-1		F7-Fl3-S3	H2	winter	CIP, TET	blaOXA-193;blaOXA-61_G-57T;gyrA_T86I;tet(O)	
	24-EP00015-1		F6*-Fl3-S3	H3	winter	CIP, TET	blaOXA-193;blaOXA-61_G-57T;gyrA_T86I;tet(O)	reared and sampled for farm 8
	24-EP00083-3		F6-Fl4-S3	H3	spring	CIP, TET	blaOXA-193;blaOXA-61_G-57T;gyrA_T86I;tet(O)	
	24-EP00097-3		F7-Fl4-S4	H2	spring	CIP, TET	blaOXA-193;blaOXA-61_G-57T;gyrA_T86I;tet(O)	
	24-EP00105-4		F6-Fl5-S3	H3	spring	CIP, TET	blaOXA-193;blaOXA-61_G-57T;gyrA_T86I;tet(O)	
	24-EP00147-4		F6-Fl5-S5	H3	summer	CIP, TET	blaOXA-193;blaOXA-61_G-57T;gyrA_T86I;tet(O)	

C. jejuni

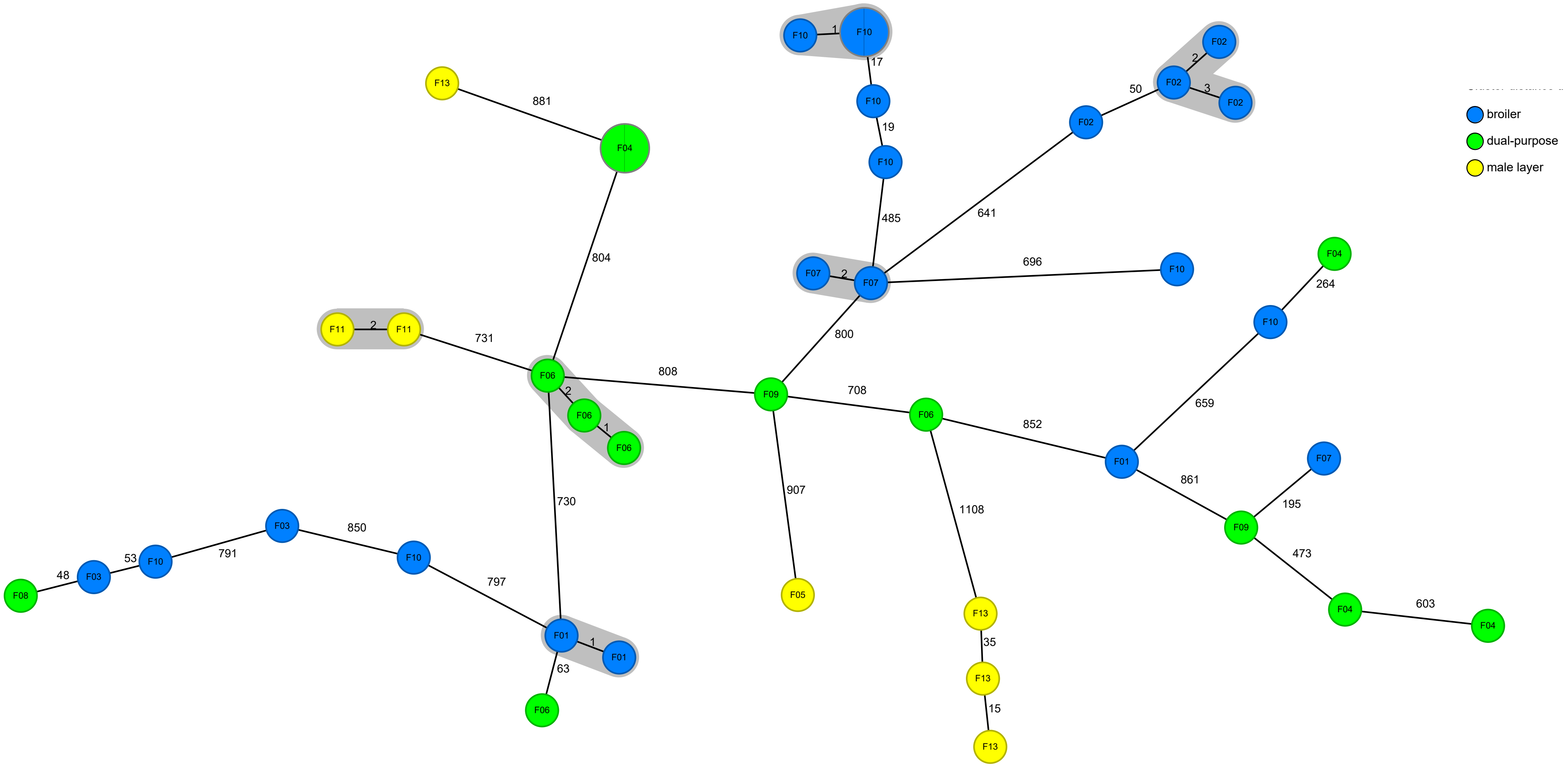

cgMLST-based minimum spanning tree for 41 *C. coli* constructed in Ridom SeqSphere + v10.5.2 with a scheme of 1,343 core loci, nodes annotated with farm number and fattening type (colour)

C. coli
